# Vaccine and antiviral drug promise for preventing post-acute sequelae of COVID-19, and their combination for its treatment

**DOI:** 10.3389/fimmu.2024.1329162

**Published:** 2024-08-09

**Authors:** Tomonari Sumi, Kouji Harada

**Affiliations:** ^1^ Research Institute for Interdisciplinary Science, Okayama University, Okayama, Japan; ^2^ Department of Chemistry, Faculty of Science, Okayama University, Okayama, Japan; ^3^ Department of Computer Science and Engineering, Toyohashi University of Technology, Toyohashi, Aichi, Japan; ^4^ Center for IT-Based Education, Toyohashi University of Technology, Toyohashi, Aichi, Japan

**Keywords:** post-acute sequelae of SARS-CoV-2 infection, PASC, long Covid, persistent viruses, vaccine, antiviral drug, mathematical model, immune response

## Abstract

**Introduction:**

Most healthy individuals recover from acute SARS-CoV-2 infection, whereas a remarkable number continues to suffer from unexplained symptoms, known as Long COVID or post-acute COVID-19 syndrome (PACS). It is therefore imperative that methods for preventing and treating the onset of PASC be investigated with the utmost urgency.

**Methods:**

A mathematical model of the immune response to vaccination and viral infection with SARS-CoV-2, incorporating immune memory cells, was developed.

**Results and discussion:**

Similar to our previous model, persistent infection was observed by the residual virus in the host, implying the possibility of chronic inflammation and delayed recovery from tissue injury. Pre-infectious vaccination and antiviral medication administered during onset can reduce the acute viral load; however, they show no beneficial effects in preventing persistent infection. Therefore, the impact of these treatments on the PASC, which has been clinically observed, is mainly attributed to their role in preventing severe tissue damage caused by acute viral infections. For PASC patients with persistent infection, vaccination was observed to cause an immediate rapid increase in viral load, followed by a temporary decrease over approximately one year. The former was effectively suppressed by the coadministration of antiviral medications, indicating that this combination is a promising treatment for PASC.

## Introduction

**Figure 1:**
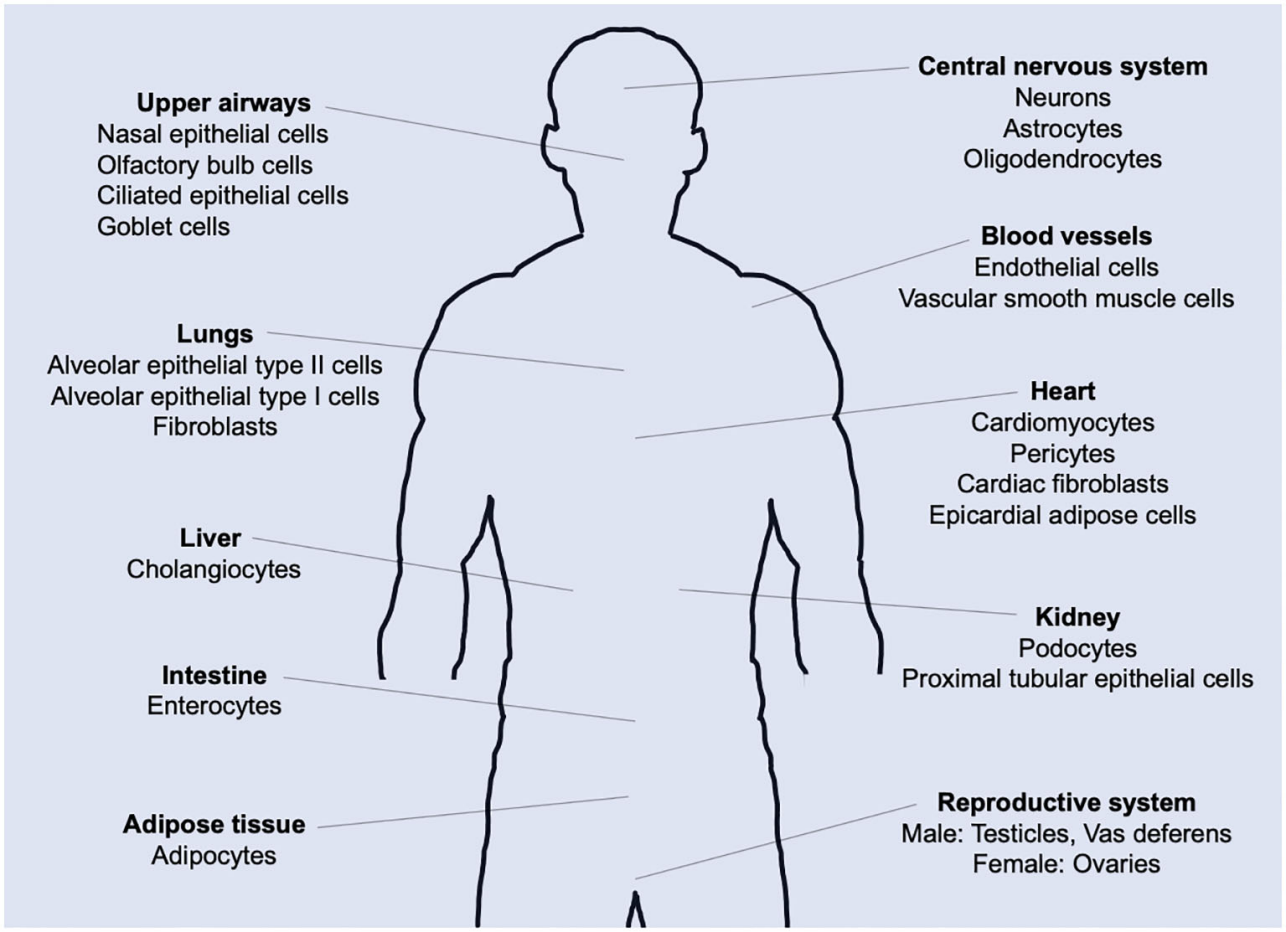
ACE2 expression in the human body. Tissue distribution of angiotensin-converting enzyme 2 (ACE2) expression and potential targets for SARS-CoV-2 infection-induced direct cytotoxicity.

Most healthy individuals recover from acute SARS-CoV-2 infection, whereas a remarkable number continues to suffer from unexplained symptoms, known as Long COVID or post-acute COVID-19 syndrome (PACS) (1–3). The World Health Organization (WHO) defines Post-Acute Sequelae of SARS-CoV-2 Infection (PASC) as a constellation of symptoms that persist or emerge anew three months after the initial SARS-CoV-2 infection, with these symptoms enduring for a minimum of two months and lacking clear attribution to other underlying causes (4). PASC is diagnosed in patients who develop severe acute COVID-19 but also in patients who experience only mild or asymptomatic cases (1). Common symptoms of PASC are fatigue, shortness of breath, and cognitive dysfunction, in addition to more than 200 reported symptoms impacting everyday functioning (4). Now, PASC is recognized as a disease with a broad spectrum of manifestations including pulmonary diseases, cardiovascular diseases, neuropsychiatric diseases, renal injury, endocrine disorders, and dysbiosis of gut microbiome (5). The diverse organ diseases associated with PASC partially reflect the fact that SARS-CoV-2 can infect a wide range of human cells (6). The receptor-binding domain (RBD) of the SARS-CoV-2 spike protein binds to the human angiotensin-converting enzyme 2 (ACE2) receptor, and the virus enters and infects host cells if the spike subunit is primed by cellular serine proteases such as TMPRSS2 and TMPRSS4 (7). As ACE2 and TMPRSS2 are expressed in a wide range of cell types (8–10), SARS-CoV-2 can infect host cells in various organs throughout the body (**Figure 1**) (11). Consequently, PASC is associated with multiple organ systems and its clinical presentation is heterogeneous and complicated, making it difficult to determine the underlying causes of the sequelae.

Different scenarios for PASC development have been discussed (12, 13), and potential hypotheses for PASC symptoms have been proposed (14–16). In a previous study, we developed a mathematical model for the immune response to SARS-CoV2 infection based on cell immunology and theoretically demonstrated the possibility of persistent infection caused by a small number of within-host viruses (17). One of the causes on developing persistent infection is attributed to the fact that infectable host cells by SARS-CoV-2 are abundant throughout the body (8–10), making it difficult for the immune system to clear the remaining within-host viruses. Potential contributors to PASC symptoms that directly or indirectly depend on persistent infection include (i) chronic inflammation caused by persistent infection in virus reservoirs, (ii) impact of unrepaired tissue injuries due to persistent viruses, (iii) chronic inflammation promoted by dysbiosis of the microbiome and the resulting microbial translocation, (iv) reactivation of other latent viral infections under immunological dysregulation caused by SARS-CoV-2 persistent infection. In the *DISCUSSION*, the validity of the potential contributors to PASC mentioned above is argued based on related clinical observations.

The aim of the present study was to develop a mathematical model of immune responses to SARS-CoV-2 infection and vaccination and to examine prevention and treatment methods for the development of PASC. To date, many mathematical models have been developed to analyze the within-host dynamics of SARS-CoV-2 infection and/or vaccination (18–47). Several mathematical modeling studies including ours have discussed the instability of virus-free equilibrium and stability of the virus co-existence equilibrium (17, 22, 25, 28, 34, 35, 39, 44, 48), mathematically supporting the clinical observations of viruses persisting to some extent within the host (49–58). Furthermore, several mathematical modeling studies have analyzed the dynamics of vaccine-induced production of SARS-CoV-2 neutralizing antibody (42, 43, 59, 60) and the effects of vaccines on preventing the aggravation of COVID-19 (25, 43, 47).

**Figure 2:**
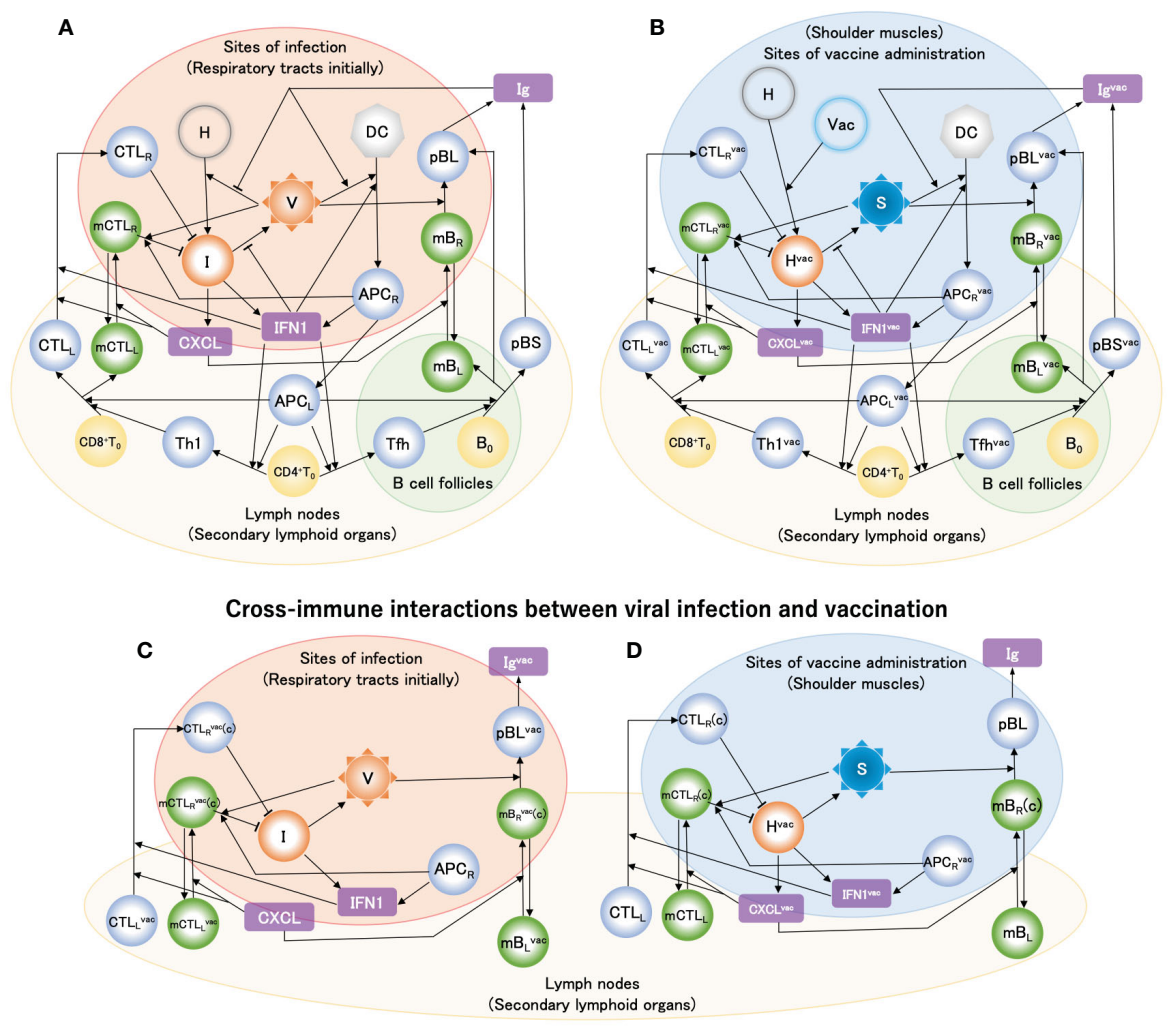
Mathematical model of host immune response to SARS-CoV-2 infection and vaccinations. Solid arrow denotes either activation or differentiation, and blunt arrow denotes inhibition. Model variables include healthy cells [H], infected cells [I], viral loads [V], dendritic cells [DC], antigen-presenting cells generated as maturating DC at infection sites [APC_R_] and in lymph nodes [APC_L_], naïve CD4^+^ and CD8^+^ T cell [CD4^+^T_0_] and [CD8^+^T_0_], naïve B cells [B_0_], type-I helper T cells [Th1], T follicular helper cells [Tfh], cytotoxic T lymphocytes in lymph nodes [CTL_L_] and infection sites [CTL_R_], short-lived and long-lived plasma B cells [pBS] and [pBL], memory CTL in lymph nodes [mCTL_L_] and infection sites [mCTL_R_], memory B cells in lymph nodes [mB_L_] and infection sites [mB_R_], type-I interferon [IFN1], chemokine receptor ligand [CXCL], and immunoglobulin [Ig]. Superscript ‘vac’ of variables [see e.g., **(B)**] indicates species generated by vaccine particles (Vac). ‘(c)’ of e.g., [mCTL_R_^vac^(c)] in **(C)** and [mCTL_R_(c)] in **(D)** indicates cells that migrated via cross interactions induced by CXCL and CXCL^vac^, respectively. **(A)** Model of immune response when viral infection occurs. **(B)** Model of immune response when a vaccine is administered. **(C)** Immune response that is added to the model shown in **(A)** when viral infection occurs after vaccination. **(D)** Immune response that is added to the model shown in **(B)** when a vaccine is administered after viral infection. In the immune response to vaccine **(B)**, the point that neither vaccine particle [Vac] nor spike protein [S] causes self-replication is most different from viral infection, while most of them in **(B)** are almost similar to those in **(A)**. The typical flows in the immune responses depicted in these figures are explained in the *METHODS*.

In the present study, we developed a mathematical model incorporating both the immune response to SARS-CoV-2 infection at the sites of infection (respiratory tracts, initially) and the vaccine at the sites of vaccine administration (shoulder muscles) (**Figures 2A, B**). In this model, cross-immune interactions arising from vaccination and viral infection at the sites of viral infection and vaccine administration, respectively, are also included (**Figures 2C, D**). By applying the model, we revealed the role of the 1^st^ and 2^nd^ doses of the primary vaccination series in antibody production and the difference in immune responses to vaccination between seronegative and seropositive persons of SARS-CoV-2. We also examined the effects of the vaccine and antiviral drugs for preventing severe disease and the development of PASC and the timing of booster vaccination to keep vaccine-induced antibody titers comparable to those of the primary series. These results provided insights into the dynamics of persistent infection and its potential contributions to PASC symptoms. Furthermore, the present analysis suggested that the co-administration of antiviral medications with vaccination was a promising approach for treating PASC patients with persistent infection.

## Results

### Baseline model appropriately reproduces the enhancement of antibody production by 2^nd^ dose of primary vaccination series

**Figure 3:**
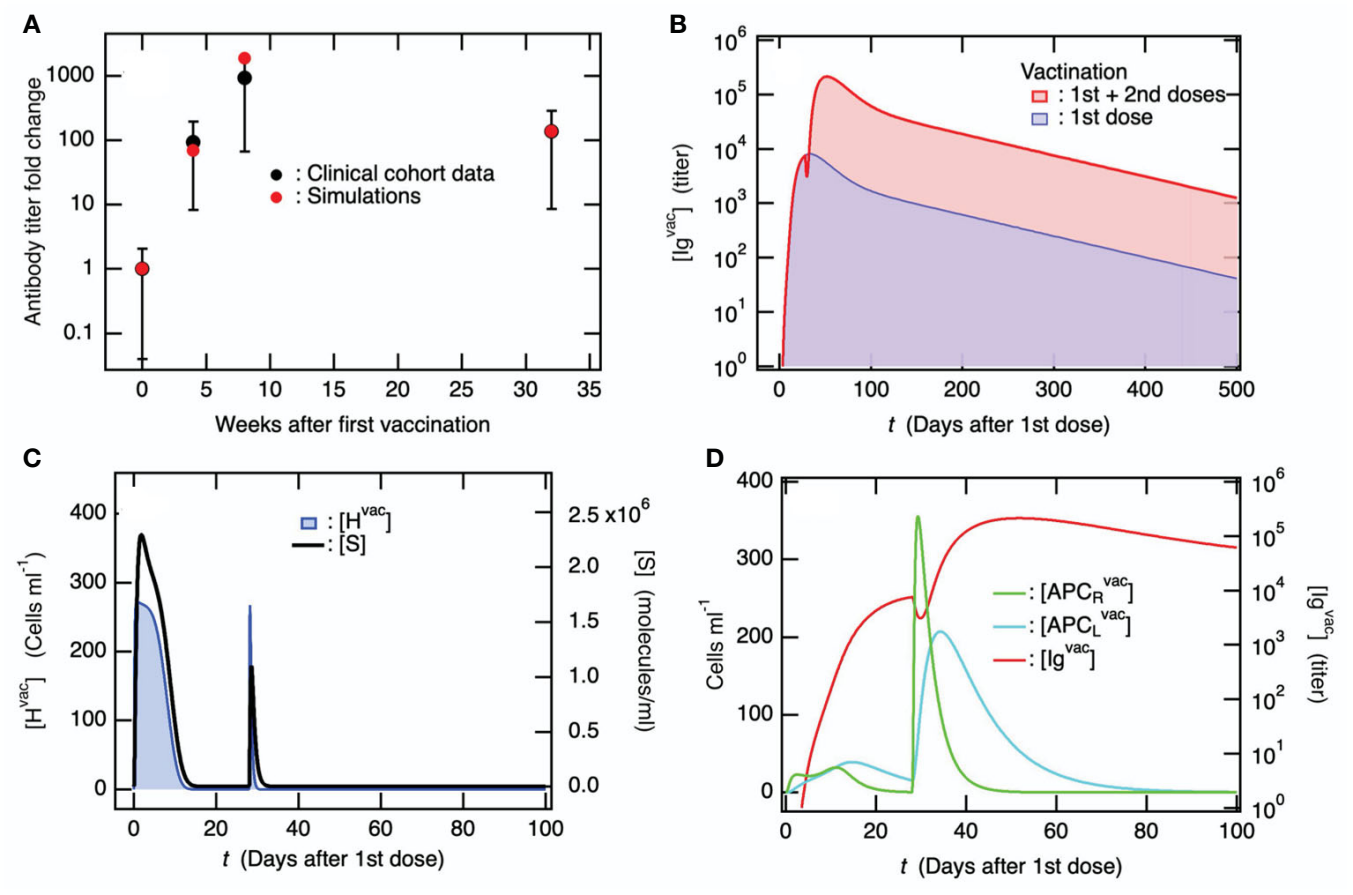
The second vaccine dose, administered following the initial dose in the primary series, effectively stimulates the production of antibodies against the coronavirus. In the simulations, the 2^nd^ vaccine dose was administrated 4 weeks after the 1^st^ dose. **(A)** Fold-change of antibody titer compared to before the 1^st^ vaccine dose upon the 1^st^ and 2^nd^ dose of the primary vaccination series. Clinical data were obtained from literature (61), where antibody titers were measured before the 1^st^ vaccine dose, before the 2^nd^ vaccine dose, 4 weeks after the 2^nd^ dose, and 6 months after the 2^nd^ dose. **(B)** Time courses of [Ig^vac^] upon only the 1^st^ vaccine dose and the 1^st^ plus 2^nd^ vaccine dose. **(C)** Time course of [H^vac^] (left axis) and [S] (right axis) upon the 1^st^ and 2^nd^ doses of the primary vaccination series. **(D)** Time course of [APC_R_^vac^] and [APC_L_^vac^] (left axis) which are compared with that of [Ig^vac^] (right axis).

Ordinary differential equations, which are schematically summarized for vaccine administration in **Figure 2B**, were solved using the initial values of the variables provided in **Supplementary Table S1** and the parameters provided in **Supplementary Table S2** (see **Supplementary Material** (SM)). The 1^st^ and 2^nd^ doses of the primary vaccination series were modeled using **Supplementary Equation S1** in the SM as two influxes of liposomes containing vaccine mRNA. The 2^nd^ dose was administered 4 weeks after the 1^st^ dose. **Figure 3A** shows the simulation results of the fold change from before the first vaccination in antibody titers before the second vaccination, four weeks after the second vaccination, and six months after the second vaccination, along with the clinical data (61). The baseline model reproduced the enhancement of antibody production by the 2^nd^ dose, and the long-term antibody titer was observed six months after the 2^nd^ dose. A comparison of the time courses of [Ig^vac^] between the 1^st^ dose and the 1^st^ dose plus 2^nd^ dose is shown in **Figure 3B**. [Ig^vac^] rapidly decreased immediately after the 2^nd^ dose and immediately increased to a maximum at ~24 days after the 2^nd^ dose. The decay constants in both cases were almost equivalent, implying a common governing factor. In **Figure 3C**, the variables of cells infected with the mRNA vaccine [H^vac^] and spike protein antigen [S] are displayed as a function of days after the 1^st^ vaccine dose. The increases in [H^vac^] and [S] after the 2^nd^ vaccine dose decreased more rapidly than those after the 1st dose because of pre-existing antibodies produced by the 1^st^ dose. Even though the clearance of the spike protein antigen is accelerated by the antibodies (**Figure 3C**), the sequence of immune responses starting from the significant increases in [APC_R_^vac^] is enhanced by the accelerated priming of DC because of the antibody-induced efficient engulfment of the antigens via the Fc receptors of DC (**Figure 3D**) (62–64). Consequently, as seen in **Figure 3D**, much higher antibody production than of the 1^st^ dose was caused by the plasma cells.

### Baseline model for immune response to SARS-CoV-2 infection develops persistent infection

**Figure 4:**
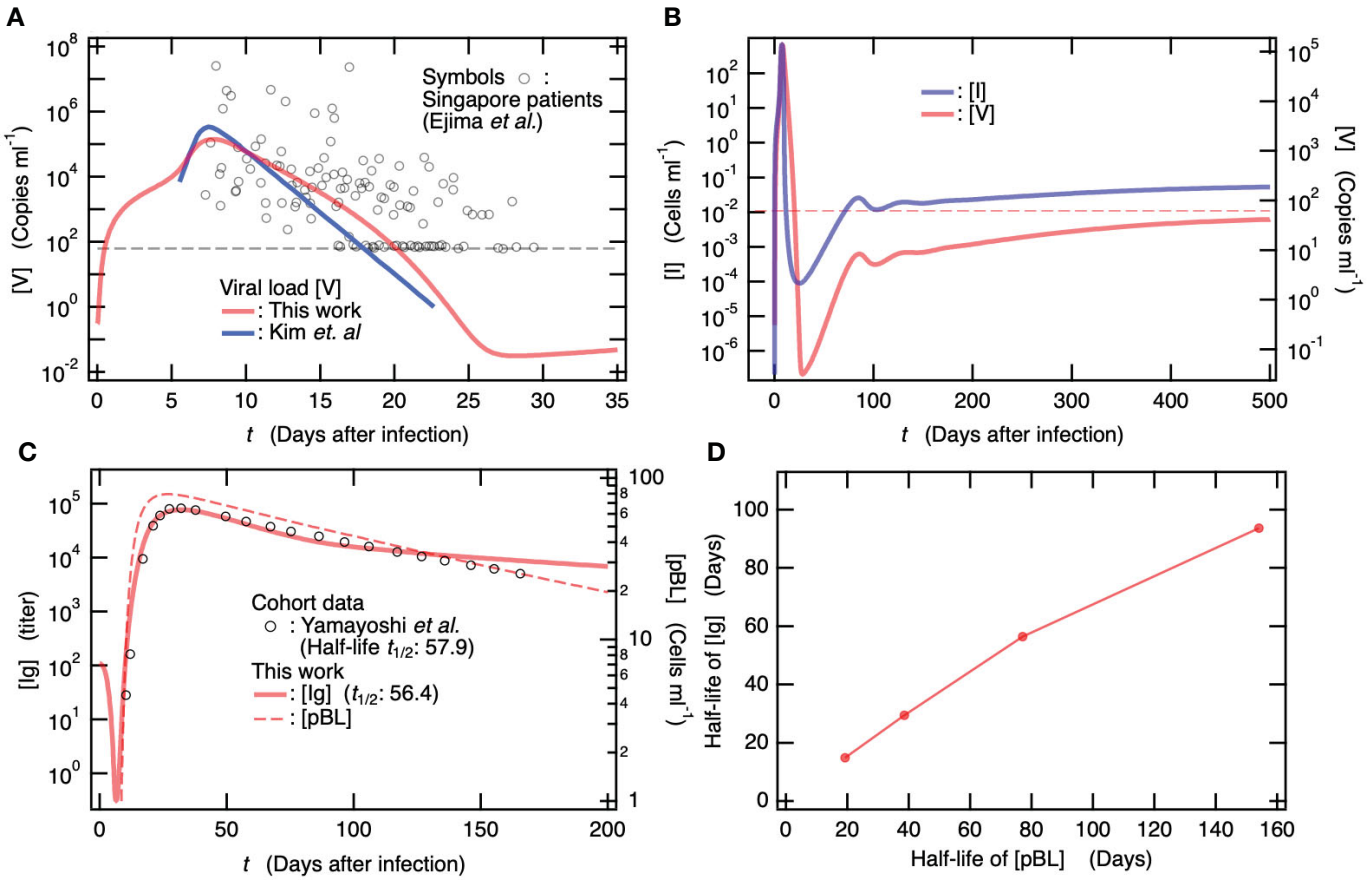
The mathematical model updated by incorporating memory T and B cells predicts persistent infection as well as the previous model without memory cells. All simulation and clinical data depicted here are for patients with neither vaccination nor viral infection. **(A)** Time course of viral load [V] by the new baseline model that was updated from our previous model (17). Symbols are viral load data for Singapore COVID-19 patients (24). For comparison, a mathematical model solution by Kim et al. (26) is also shown. The dashed horizontal line indicates viral detection limit. **(B)** Longitudinal change of [I] (left axis) and [V] (right axis). The dashed horizontal line indicates viral detection limit. **(C)** Comparison of [Ig] obtained from the baseline model against longitudinally clinical data for symptomatic patients (65). Time course of [pBL] is also shown on the right axis. Half-life $t_{1/2}$ of Ig determined by the baseline model is 56.4 days, which is quantitatively consistent with the clinical data $t_{1/2}$ of 57.9 days (65). **(D)** Half-life of [Ig] vs. half-life of [pBL]. The latter was determined by $t_{1/2}=\text{ln}\left(2\right)/\delta_{pBL}$ , where only the death rate of pBL $\delta_{pBL}$ in the baseline model was changed in the simulations for determining [Ig].

Ordinary differential equations, which are schematically summarized for viral infections in **Figure 2A**, were solved using the initial values of the variables in **Supplementary Table S1** and the parameters provided in **Supplementary Table S2**. Memory T and B cells were incorporated into the newly developed mathematical model (**Figure 2A**). Short- and long-lived plasma cells were also considered. **Figure 4** shows the variables in the solution obtained for the baseline model, plotted as a function of the number of days after infection. The viral load [V] was compared against the model (blue line) which Kim et al. determined using available viral load data (26), in addition to the data for Singapore COVID-19 patients (24) (**Figure 4A**). Hereafter, the time to symptom onset after infection, which was mathematically determined as ~5.62 days by Ejima et al. using viral load data (24), was utilized for comparison with clinical data. The dashed horizontal lines in **Figures 4A, B** indicate the viral detection limits. The time course of [V] obtained from the present model (**Figure 4A**) was similar to that of the previous model (17). Specifically, the long-term behaviors of [V] and [I] (**Figure 4B**) were qualitatively consistent with those obtained from the previous model (17), and a persistent infection caused by the remaining within-host viruses was found. Similar to the previous model, the linear stability analysis demonstrated that the virus-free equilibrium was unstable (**Supplementary Table A1** in the Supplementary Material), whereas the virus coexistence equilibrium was stable (**Supplementary Table A2** in the Supplementary Material), even though the present model included memory immune cells and long-lived plasma cells. In contrast, it was shown by the linear stability analysis that a virus-free equilibrium became stable when the viral infection rate $\pi_{I}$ and viral production rate $\pi_{V}$ were reduced from the baseline model (**Supplementary Table A3** in the Supplementary Material).

In **Figure 4C**, the time courses of [Ig] (left axis) and [pBL] (right axis) are shown as functions of the number of days after infection. For comparison, the clinical data of antibody titers for symptomatic patients (65) are shown (**Figure 4C**). The simulation result for [Ig] was quantitatively consistent with the clinical data (65). In addition, the half-life $t_{1/2}$ of [Ig] determined from the present simulation was 56.4 days and was quantitatively consistent with $t_{1/2}$ which was determined from clinical data (57.9 days) (65). As shown in **Figure 4C**, the asymptotic behavior of [Ig] seems to depend on that of the long-lived plasma cells [pBL]. To figure out the long-term relation between [Ig] and [pBL], the half-life of [Ig] was calculated with variability only in the death rate of pBL $\delta_{pBL}$ in the baseline model. **Figure 4D** shows the half-life of [Ig] vs. that of [pBL], where the latter was determined using the relation $t_{1/2}=\text{ln}\left(2\right)/\delta_{pBL}$ . This correlation between [Ig] and [pBL] indicates that the long-term behavior of [Ig] depends mainly on antibody production by long-lived plasma cells.

The memory B cells, mB_R_, are located in the infected regions, awaiting reinfection. Upon recognizing antigens via specific receptors (66), they transform into pBL (67), which produce antibodies. The memory cytotoxic T lymphocytes, mCTL_R_, also reside in the infected regions in anticipation of reinfection and reactivate upon recognition of antigens with the assistance of APC_R_ (68) to kill infected cells. To investigate the effect of these memory cells on the virus co-existence equilibrium, the steady state value of [V] was calculated for the baseline models without the memory T and B cells (see **Supplementary Table A4** in the Supplementary Material). Neither the memory T cell nor the memory B cell were found to exhibit any apparent effect on the steady state value of [V] at the virus co-existence equilibrium.

### Immune response to vaccination is much stronger in subjects infected previously

**Figure 5:**
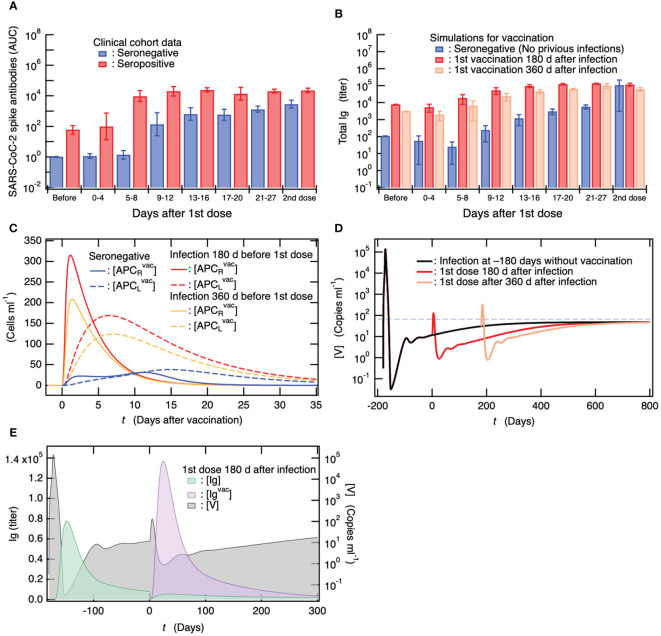
Antibody titers in individuals who are already seropositive are considerably higher than those in seronegative individuals, and the efficiency of antibody production in seropositive individuals after the first vaccine dose is comparable to that observed in seronegative individuals after receiving the second dose of the primary vaccination series. **(A)** Clinical data on antibody titers over time after a single dose of vaccine for seronegative vaccinees without previous infection and for seropositive persons with previous infection (69). Antibody titers of both vaccinees 4 weeks after 2^nd^ vaccine dose are also shown. **(B)** Comparison of simulation results of total Ig titer ([Ig] + [Ig^vac^]) among seronegative vaccinees without previous infection and seropositive vaccinees who were infected 180 and 360 days before a single dose. The total Ig titers of these vaccinees 4 weeks after 2^nd^ vaccine dose when the 2^nd^ dose was administrated 4 weeks after the 1^st^ dose are also shown. **(C)** Comparison of APC activations upon a single dose of vaccine among seronegative persons as well as among seropositive persons who were infected 180 and 360 days before the single dose. The strength of APC activation depends on antibody titer upon the vaccination because DC is activated by Ig-binding to its Fc receptor and efficiently engulfs antigens bound to Ig (62–64). **(D)** [V] of seropositive persons upon a vaccination at 180 and 360 days from infection as a function of days from 180 days after infection. For comparison, [V] for persons without vaccination is also shown together with the viral detection limit (dashed horizontal line). **(E)** Correlation between antibody titer and remaining within-host viral load.

A cohort study investigated the antibody response to 1^st^ vaccine dose in seropositive persons with previous SARA-CoV-2 infection (less than one year after the infection) (69). The antibody titers of the seropositive vaccinees have been found to be ~15 times higher than those of seronegative vaccinees without previous infection 4 weeks after the 1^st^ vaccine dose (**Figure 5A**) (69). Notably, the antibody titers of the seropositive vaccinees at this point were already higher than those at 4 weeks after the seronegative individuals were vaccinated with 2^nd^ dose of the primary vaccination series (**Figure 5A**) (69). Here, for comparison with the clinical data, total Ig titers, given as the sum of [Ig] and [Ig^vac^], were calculated when 1^st^ vaccine dose was administered to seropositive individuals who had been infected 180 and 360 days before the 1^st^ dose. The results obtained for seropositive vaccinees are shown in **Figure 5B**, together with those for seronegative vaccinees without previous infection. The total Ig titers of the seropositive vaccinees were always higher than those of the seronegative vaccinees, and the antibody production efficiency of the seropositive vaccinees after the 1^st^ vaccine dose was more than or comparable to that when the seronegative individuals were vaccinated with 2^nd^ dose of the primary vaccination series (**Figure 5B**); thus, these results are consistent with the clinical data (**Figure 5A**).

In the baseline model, as shown in **Figure 4**, the virus coexistence equilibrium is in a stable steady state after infection. Thus, in the vaccine administration to seropositive persons shown in **Figure 5B**, a small amount of virus remains in the vaccinees, and persistent infection occurs at the time of vaccination. Therefore, it is possible that the highly efficient antibody production in seropositive vaccinees reflects viral persistence. As shown in **Figure 5B**, the total Ig titers of seropositive vaccinees infected 180 days before vaccination were always higher than those of seropositive vaccinees infected 360 days before vaccination. However, the remaining viral load of the seropositive persons who had been infected 180 days before vaccination at the time of vaccination was lower than that of the seropositive persons who had been infected 360 days before vaccination (**Figure 4B**). This indicates that the influence of persistent viruses on the enhanced antibody production of seropositive vaccinees is less important than that of preexisting antibodies.

To determine the difference in the immune responses to vaccination between the seronegative and seropositive vaccinees, the time courses of [APC_R_^vac^] and [APC_L_^vac^] for seropositive vaccinees who were infected 180 and 360 days before vaccination, as well as for the seronegative vaccinees, are shown as a function of the number of days after vaccination (**Figure 5C**). In the seronegative vaccinees, the maturation of DC working as APC was not sufficient, and the migration of APC to the lymph nodes was delayed. On the other hand, in the seropositive vaccinees, the activation of APC was enhanced by within-host pre-existing antibodies, and the APC quickly migrated to the lymph nodes to present the antigens to T and B cells; thus, antibody production is expected to be more efficient. Notably, the enhancement of antibody production by pre-existing antibodies that had been produced upon infection in seropositive vaccinees was sufficiently effective 360 days after infection (**Figure 5B**).

### Vaccination temporally reduces persistent viruses but cannot rescue from virus co-existence equilibrium

The reason for expecting a therapeutic effect of vaccine administration in patients who develop PASC is the possibility of persistent infection behind PASC symptoms. Several cohort studies have investigated how vaccine therapy for people with PASC affects the range and severity of symptoms (70–76). Among the participants experiencing PASC symptoms, 22–58% reported improvements in symptoms, 18–31% reported deterioration, and 62–71% reported no changes (72, 74). Furthermore, among PASC symptoms reported before vaccination, 17–23% improved, 6–21% worsened, and 62–71% remained unchanged (71, 75). This heterogeneity in the response of PSAC patients to vaccines has been attributed to the complexity of the underlying causes of PASC, including persistent infection.

**Figure 5D** shows the time course of viral load after vaccination in seropositive vaccinees who were infected 180 and 360 days before vaccination and developed persistent infection. For comparison, the time course of [V] in the baseline model of unvaccinated patients is shown in **Figure 5D**. In both seropositive vaccinees, a rapid increase in [V] upon vaccine administration and a temporary decrease for more than 1 year were observed, while the stability of the virus co-existing equilibrium was not affected by the vaccination (also see **Supplementary Table A5** in the Supplementary Material). In **Figure 5E**, the time courses of [Ig] and [Ig^vac^] produced via infection 180 days before vaccination and via vaccination are shown as a function of the number of days after vaccination. For comparison, the time course of [V] is shown on the right axis of **Figure 5E**. It was found that [Ig] rapidly decreased upon vaccination, and in parallel, [V] rapidly increased with the depletion of [Ig]. Subsequently, [Ig^vac^] increased such that viral production by persistently infected cells was temporarily suppressed by the transient increase in [Ig^vac^]. However, [V] asymptotically reached a virus co-existence equilibrium, which was equivalent to that before vaccination (**Figure 5D**).

Based on the dynamics of the viral load following vaccination, the clinically observed treatment effects of vaccination on patients with PASC mentioned above can be interpreted as follows. If the four potential contributors to PASC arising directly and indirectly from persistent infection, which are proposed in the *INTRODUCTION*, are partially eliminated or alleviated during the long-term but transient reduction in [V], the participants will be diagnosed as having improved or will feel an improvement by themselves. However, if the four potential contributors to PASC are further developed by the rapid transient rise in [V] following vaccination, the participants will be diagnosed with deterioration or may feel worsening by themselves. Alternatively, since vaccination cannot affect the stability of the virus co-existence equilibrium and the immune state asymptotically returns to the virus co-existence equilibrium similar to that before vaccination, PASC symptoms might not improve depending on the individual causes. To use vaccination as a treatment for PASC, efforts should be made to reduce the risk of vaccine-mediated exacerbation as much as possible. The potential of combining it with antiviral medications to reduce this risk is examined later.

### Duration of vaccine efficacy and its impact on virus co-existence equilibrium

**Figure 6:**
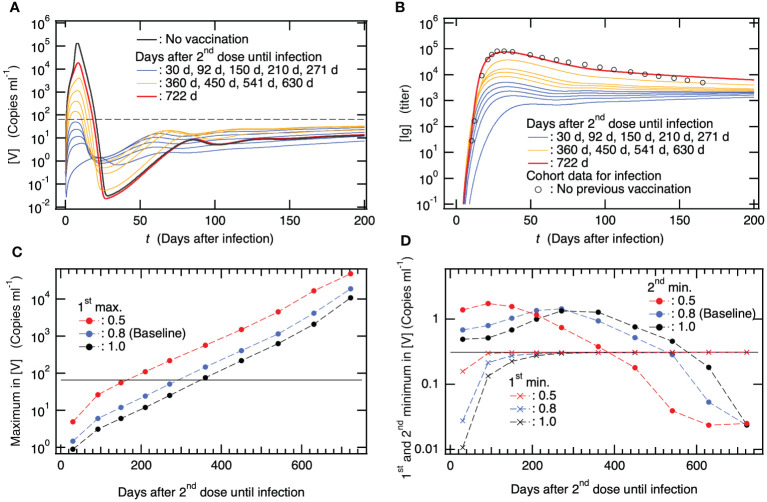
The reported clinical efficacy of the vaccine in preventing the development of Post-Acute Sequelae of SARS-CoV-2 (PASC) is primarily associated with its ability to prevent severe disease following vaccination. **(A)** Time course of viral load [V] along with infection after several months from 2^nd^ dose of primary vaccination series as a function of days after the infection. For comparison, [V] for patients with neither previous vaccination nor viral infection is also shown together with the viral detection limit (dashed horizontal line). **(B)** Time course of [Ig] when patients were infected several months after 2^nd^ dose of primary vaccination series as a function of days after the infection. For comparison, clinical data of antibody titer due to viral infection in patients with neither previous vaccination nor viral infection (65) is also shown. **(C)** Maximum in [V] vs. days after 2^nd^ vaccine dose until infection. In addition to the baseline model where reduction rate in crossing interactions between viral infection and vaccination is set to be $\theta_{cross}=0.8$ (see **Supplementary Table S2** in the SM), the results from two models using $\theta_{cross}=0.5\text{ and }1.0$ are also shown. The horizontal line indicates the viral detection limit. **(D)** The first and second minimums in [V] vs. days after 2^nd^ vaccine dose until infection. The result from the baseline model with $\theta_{cross}=0.8$ and two models using $\theta_{cross}=0.5\text{ and }1.0$ are also shown. The horizontal line indicates the initial value of [V] at t=0 (see **Supplementary Table S1** in the SM).

Because an effective treatment for PASC has not yet been established, a possible measure is to prevent the development of PASC symptoms. Currently, vaccination is the most effective means of preventing PASC development. Several cohort studies have reported that vaccination before SARS-CoV-2 infection is associated with a decreased prevalence of PASC compared to no vaccination (77–81). To clarify the mechanism of action of PASC prevention by vaccination, we examined how the primary vaccination series affected the dynamics of SARS-CoV-2 infection with varying days after the 2^nd^ vaccine dose until viral infection. **Figures 6A**, **5B** show the time courses of [V] and [Ig] as functions of the number of days after infection, respectively, when a patient was infected several weeks after the 2^nd^ dose of the primary vaccination series. For comparison, [V] for the baseline model without vaccinations and the clinical data for [Ig] without previous vaccinations are shown in **Figures 6A, B**, respectively. For instance, in the case of patients who were infected 30–271 days after the 2nd vaccine dose, [V] initially decreased after infection, followed by an increase (as depicted by **Supplementary Figure S1** in the **Supplementary Material**). Subsequently, it reached its first maximum and then gradually decreased towards a second minimum, eventually asymptotically increasing toward a steady-state equilibrium with the virus, accompanied by minor oscillations (**Figure 6A**). Notably, the maximum [V], even for the patient who was infected 271 days after the 2^nd^ vaccine dose, was less than the viral detection limit (dashed horizontal line in **Figure 6A**). However, the primary series of vaccinations did not eliminate the stable coexistence equilibrium with the virus (**Supplementary Table A6** in the SM). Nevertheless, the possibility of a complete cure while avoiding the development of persistent infection would remain if viruses were completely eliminated from the host around the second minimum in [V] in the same stochastic manner as pointed out in our previous study (17). Consistent with the mild viral production (**Figure 6A**), as seen in **Figure 6B**, the antibody production upon infection in these patients was not extensively activated because large amounts of antibodies [Ig^vac^] produced by the vaccinations had already existed in the host.

With an increase in the number of days after the 2^nd^ vaccine dose until infection, the maximum in [V] and [Ig] values increased (**Figures 6A, B**). In patients who were infected 2 years after the 2^nd^ vaccine dose, viral production was obviously suppressed by the host immune response compared to that in patients without vaccination (**Figure 6A**), whereas antibody production was almost equivalent to that in patients without vaccination (**Figure 6B**). If the patients benefited from the primary vaccination series 2 years before the infection to prevent severe disease, the mechanism was attributed to the immune response of memory T and B cells generated by the vaccinations. Specifically, as shown in **Figure 2C**, memory T cells, that is, mCTL_R_^vac^(c), kill infected cells, and memory B cells, that is, mB_R_^vac^(c), immediately transform into pBL^vac^ and efficiently increase [Ig^vac^]. It was impossible to eliminate the stable coexistence equilibrium with viruses, even though the host possessed large amounts of antibodies as well as memory T and B cells, due to vaccinations. In contrast, vaccination has been found to be effective in preventing severe diseases.

To show the above-mentioned effects of vaccination quantitatively, the maximum in [V] and the first and second minima in [V] are plotted in **Figures 6C, D**, respectively, as a function of the number of days after 2^nd^ vaccine dose until infection. In these figures, in addition to the results from the baseline model where the reduction rate for crossing immune interactions between viral infection and vaccination is set to be $\theta_{cross}=0.8$ , the results from two models using $\theta_{cross}=0.5\text{ and }1.0$ are also shown. $\theta_{cross}$ indicates, for example, the difference between the neutralizing ability of virus by antibodies that are produced by viral infection and vaccination. Thus, if the neutralizing ability by antibody produced by viral infection is 1, that produced by vaccination is $\theta_{cross}$ . To closely look at the 1^st^ minimum in [V], the time courses of [V] for the model using $\theta_{cross}=1.0$ are shown by **Supplementary Figure S1** in the SM as an example. The longer the days after 2^nd^ vaccine dose until infection, the larger the maximum [V] (**Figure 6C**). Varying $\theta_{cross}$ changes the maximum [V], while the trend of increasing [V] holds, indicating that the risk of severe disease increases upon increasing the number of days after 2^nd^ vaccine dose without depending on $\theta_{cross}$ . The 1^st^ minimum [V] raises with increasing days after 2^nd^ vaccine dose until infection for all the cases of $\theta_{cross}$ (**Figure 6D**). This implies that the possibility of stochastically achieving a complete cure before increasing [V] after infection decreases as the number of days after vaccination increases. On the other hand, the 2^nd^ minimum [V] decreases with increasing days after 2^nd^ vaccine dose when patients are infected more than ~300 days after 2^nd^ dose for all the cases of $\theta_{cross}$ (**Figure 6D**). This implies that the probability of stochastically breaking down the development of persistent infections around the 2^nd^ minimum increases as the number of days after vaccination increases. However, the values of 2^nd^ minimum [V] were not remarkably lower than those without vaccination; thus, this possibility might be small. Taken together, these results suggest that the vaccine effect on preventing the development of clinically reported PASC is mainly attributed to the prevention of severe disease via suppression of viral production due to vaccinations rather than stochastic blocking of the development of persistent infection.

### Effectiveness of antiviral drug administration in acute phase for PASC prevention and limited therapeutic impact of single-drug prescriptions during PASC development

**Figure 7:**
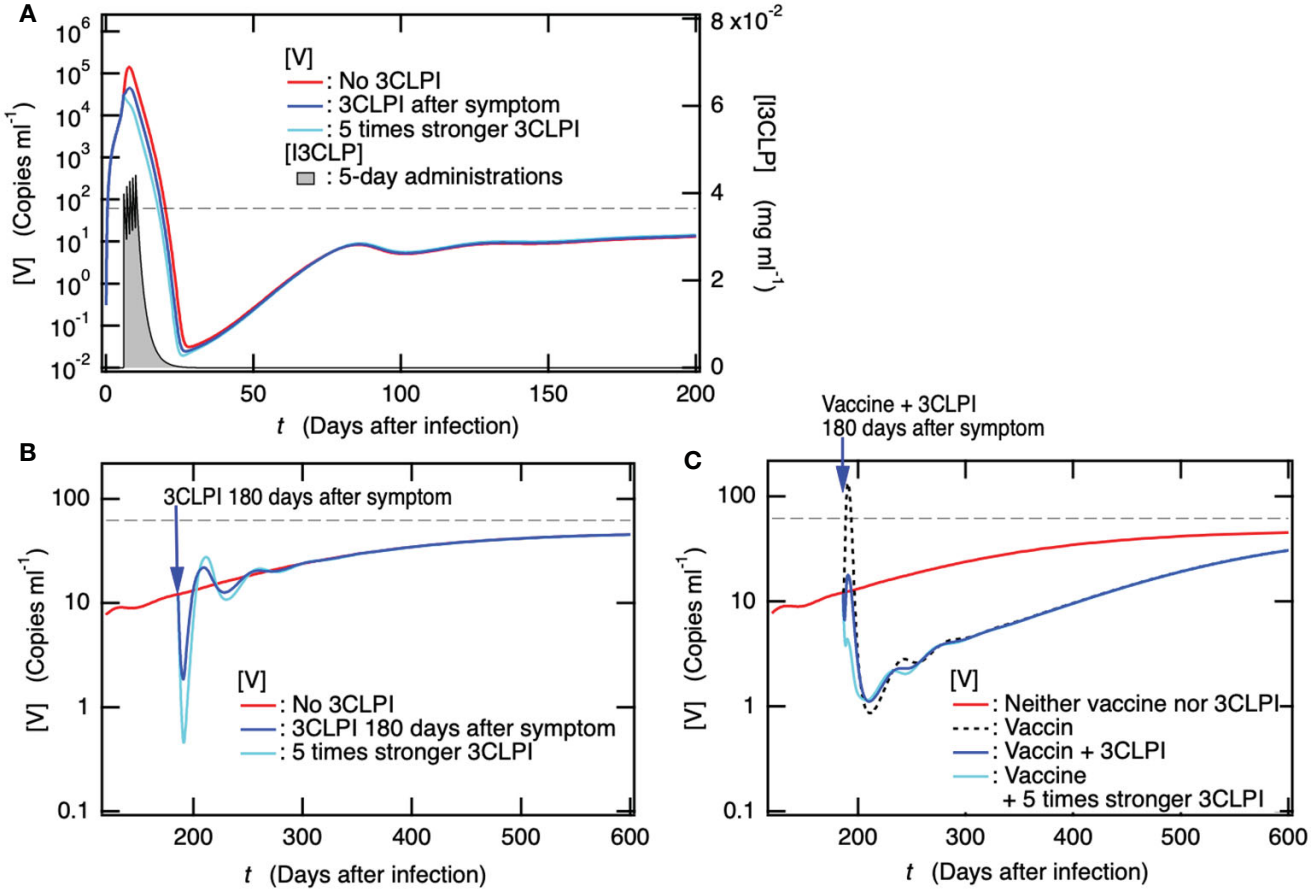
Administration of a 3C-like protease inhibitor (3CLPI) in the acute phase suppresses viral replication, thereby preventing the onset of PASC, but the antiviral-drug prescriptions alone after the development of PASC are not expected to have a marked therapeutic effect. **(A)** Time course of [V] for patient with 3CLPI administration the day after symptom onset, i.e., six days after the infection in the present case. The concentration of 3CLPI [I3CLP] is also shown for the right axis as a function of day after infection. **(B)** Time courses of [V] for patients with 3CLPI administration 180 days after symptom onset, i.e., 186 days after infection. The cases for patients with five times stronger 3CLPI administration and without 3CLPI administration are also shown. **(C)** Time course of [V] for patient with both a single dose of vaccine and 3CLPI administration 180 days after symptom onset. For comparison, the cases for patients with both five times stronger 3CLPI administration and single dose of vaccine, with only single dose of vaccine, and with neither vaccination nor 3CLPI administration are also shown.

In addition to the fact that vaccinations cannot eliminate the stable coexistent equilibrium with viruses, since the preventive effect of the vaccine confers only partial protection against PASC symptoms, additional mitigation strategies are necessary to reduce the long-term health consequences of SARS-CoV-2 infection. A cohort study has reported that the antiviral treatment with a 3C-like protease inhibitor (3CLPI) in the acute phase is associated with 26% less risk of PASC, 47% less risk of post-acute death, and 24% less risk of post-acute hospitalization (82). To reveal the mechanism of preventing PASC by antiviral drugs, the time course of [V] for patients with 3CLPI administration on the day after symptom onset, i.e., six days after the infection, was examined because symptom onset after infection was 5.6 days (24) (**Figure 7A**). For all cases of 3CLPI administration, 5-day series administration was applied using **Supplementary Equation S2** in the SM. For comparison, the case of antiviral drugs, in which the inhibition of viral production was five times stronger than that in the normal case, was also examined. As shown in **Figure 7A**, the administration of antiviral drugs reduced the maximum viral production during the acute phase and the minimum thereafter, whereas no marked effect on the long-term dynamics of [V], including persistent infection, was observed. Therefore, the antiviral drug-mediated influences on clinically observed PASC symptoms should be attributed to (1) the prevention of severe disease due to suppression of viral production (2), the stochastic prevention effect of preventing the development of persistent infection, or (3) both.

Next, we examined how antiviral drugs affected persistent infections to gain information on the influence of antiviral drug administration on PASC. **Figure 7B** shows the time course of [V] when the antiviral drug was administered to patients who developed PASC along with a persistent infection 180 days later. Even in the case of five times stronger 3CLPI, the duration at which the suppression of viral production was observed was too short to recover from PASC, and the time course of [V] promptly returned to the state without 3CLPI administration. Thus, antiviral drug prescriptions alone are not expected to have as much of a therapeutic effect on PASC symptoms as vaccine administration. We then examined how the concurrent administration of the antiviral drug and vaccine affected the rapid transient increase in [V] upon vaccine administration (**Figure 5D**), which might aggravate PASC symptoms. **Figure 7C** shows the time courses of [V] when the vaccine and antiviral drug were concurrently administered to patients who developed PASC along with persistent infection 180 days after infection. The concurrent administration of antiviral drugs completely suppressed the rapid transient rise in [V] upon vaccine administration, while it did not affect the subsequent temporary decrease in [V] appearing for more than 1 year. As illustrated in **Figure 5E**, the depletion of [Ig] results in a rapid transient increase in [V] as [Ig] is consumed by the spike protein antigen produced by the vaccination. The potential contributors to PASC discussed in the introduction could be further developed by the transient increase in [V] following vaccination. In contrast, the co-administration of the antiviral drug successfully compensated for the depletion of [Ig] so that the transient increase in [V] was completely suppressed. Therefore, the combination of these may work as a beneficial treatment for PASC, reducing the risk of deterioration that was observed in the case of vaccination alone (**Figure 5D**) and increasing the possibility of improvement in PASC. For your information, the 1.5-fold increase in vaccine dose along with the antiviral drug administration had no apparent effect on the transient decrease in [V] (**Supplementary Figure S2** in the SM).

### Antibody production efficiency upon a booster dose of vaccine strongly depends on the number of days between the last and booster dose

**Figure 8:**
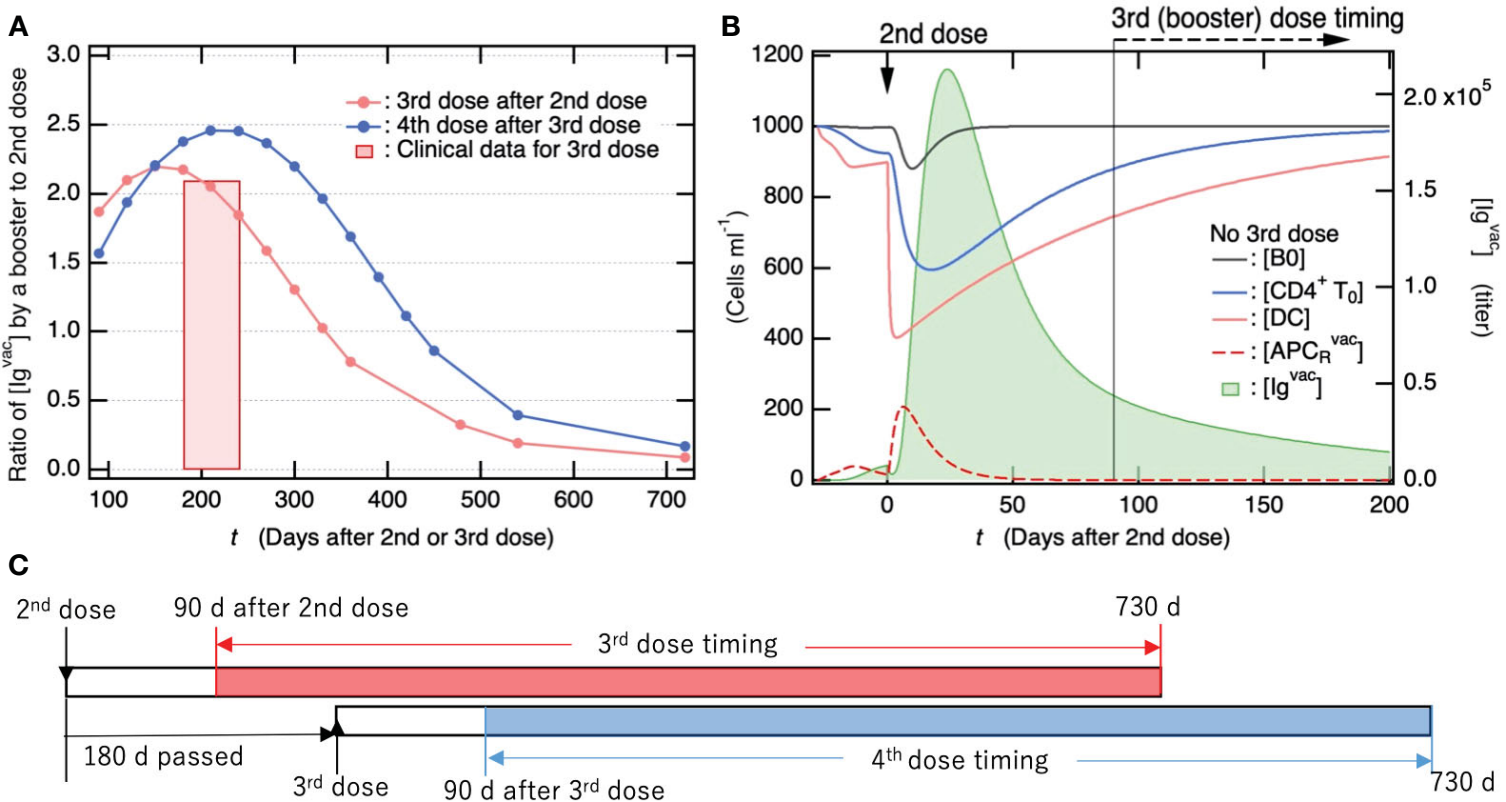
Antibody titers produced by vaccine booster doses following primary vaccination series reach more than two-fold higher than those after the 2^nd^ dose of the primary series. **(A)** Fold-change of antibody titer increased by 3^rd^/4^th^ vaccine dose compared to antibody titer upon 2^nd^ dose of primary vaccination series. The bottom axis indicates the timing of 3^rd^/4^th^ vaccine dose as the number of days after 2^nd^/3^rd^ dose. In the case study of the 4^th^ vaccine dose, the timing of the 3^rd^ dose was set to 180 days after the 2^nd^ dose of primary series. The antibody titers shown here were commonly measured 28 days after vaccine dose. For comparison, clinical data for the fold-change of antibody titer 29 days after the 3^rd^ vaccine dose (83) are also shown, where the periods until 3^rd^ dose from 2^nd^ dose were in between 6 and 8 months. **(B)** Time course of population for immune cells that are involved in antibody production (left axis) and of [Ig^vac^] (right axis) shown as a function of days after 2^nd^ dose of primary vaccination series. **(C)** Summary of time schedule for the third (fourth) vaccine dose shown in 7a.

As shown in **Figure 6A**, if individuals were infected two years after the 2^nd^ dose of the primary vaccination series, the effect on avoiding severe disease was not sufficient to fully avoid the development of PASC. Therefore, frequent and regular booster vaccine doses are required for sufficient antibody production. To gain insights into effective vaccination planning, we examined how the maximum antibody titer upon vaccination changed with varying timing of 3^rd^ and 4^th^ vaccine doses following the 2^nd^ dose of the primary vaccination series. **Figure 8A** shows how the fold-change in antibody titer upon the 3^rd^ and 4^th^ vaccine doses compared to the antibody titer upon the 2^nd^ dose of the primary vaccination series depends on the interval between the last and subsequent vaccine doses. The bottom axis indicates the timing of 3^rd^ and 4^th^ vaccine doses as the number of days after the 2^nd^ and 3^rd^ doses until the booster dose, respectively. In the case study of 4^th^ vaccine dose, the timing of 3^rd^ dose was set at 180 days after 2^nd^ dose of the primary vaccination series. The antibody titers upon 3^rd^ vaccine dose were quantitatively consistent with the clinical data (83), as indicated by the red bar in **Figure 8A**. In the 3^rd^ vaccine dose, the duration of less than 160 days from the 2^nd^ dose until 3^rd^ dose was slightly too short to obtain maximum antibody production. On the other hand, after the maximum antibody titer (approximately 160 days after the 2^nd^ dose), the longer the period between vaccine doses, the smaller the antibody titer.

To elucidate the factors influencing the fluctuations in antibody titers, we examined the temporal trends of immune cell populations engaged in antibody production over time following the second vaccine dose (as depicted in **Figure 8B**). Specifically, [CD4^+^T_0_] and [DC] exhibited a rapid decline after the second vaccine dose and had not fully recovered even by the time of the third booster dose administration (90 days post the 2^nd^ dose). The increase in vaccine-induced antibody production from 90 to 160 days was attributed to the recovery of these immune cells (**Figure 8B**). However, the decrease in vaccine-induced antibody production with increasing the number of days from ~160 days is attributed to a decrease in Ig^vac^-induced activation of DC and resulting APC_R_^vac^. This is because the preexisting [Ig^vac^] decreased with the number of days after the 2^nd^ dose (**Figure 8B**). Notably, when the interval between vaccine doses exceeds two years, the antibody titer produced by a subsequent vaccination is anticipated to be similar to that observed after the initial dose of the primary vaccination series and markedly lower than the levels achieved after the second dose. To achieve the same level of antibody titer as the 2^nd^ dose of the primary vaccination series, it is necessary to continue with the vaccine booster dose at least once a year.

## Discussion

In the *INTRODUCTION*, we proposed potential contributors to PASC symptoms that depend directly or indirectly on persistent infection. Specific clinical observations supporting the validity of the proposed potential contributors to PASC are presented below. A growing number of studies have shown that some patients infected with SARS-CoV-2 do not successfully clear the virus over long periods. Several studies have identified the persistence of SARS-CoV-2 RNA in the olfactory neuroepithelium (49), gastrointestinal tract (50, 51), feces (52), blood plasma (84), and specific cell types in the lungs (53). One of these studies indicated that anti-SARS-CoV-2 memory B cells display clonal turnover at 6.2 months after infection and that the memory B cell response and resistance of antibodies produced against RBD mutations evolve in a manner that is consistent with the persistence of the RNA antigen (50). Furthermore, persistent spike antigens have been detected in plasma samples from patients with PASC (54, 84) and specific lung cell types (53). Other studies have shown the persistence of antigen-specific CD8^+^ T-cell responses (55), activation of CD8^+^ T-cells with effector cytotoxic profiles (56), and persistent depletion of dendritic cells (57, 58). Persistent infection of host cells by residual within-host viruses and viral replication in infected cells should underlie these clinical observations.

Changes in the bacterial, fungal, and viral gut microbiomes have been observed as a consequence of SARS-CoV-2 infection (85, 86). A study of the long-term effects of SARS-CoV-2 infection on gut microbiota reported that patients with PASC exhibit gut microbiome compositions remarkably different from uninfected controls six months after virus clearance had been assessed via PCR (87). Alterations in the gut microbiota composition among patients with PASC enable several opportunistic pathogen populations, including *Clostridium innocuum* and *Actinomyces naeslundii* to gain a foothold and weaken the anti-inflammatory bacterial population, including butyrate-producing bacteria associated with adverse secondary outcomes (i.e., fatigue and hair loss) (87). A systems biology study provided evidence that severe COVID-19 is associated with disrupted intestinal barrier integrity, microbial translocation, and intestinal dysfunction (88). SARS-CoV-2 can infect the gut cells (89); thus, intestinal disruption can be caused directly by SARS-CoV-2 infection and/or indirectly by systemic inflammation due to infection and lung injury. Notably, unrepaired tissue injuries in the gastrointestinal tract due to persistent viral infections can cause persistent dysbiosis of the gut microbiome and long-term microbial translocation. Therefore, long-term alterations in the gut microbiota composition due to disrupted intestinal barrier integrity, which is not repaired by persistent viral infection, along with the resulting persistent microbial translocation-mediated chronic inflammation, should contribute to PASC symptoms (90).

Other latent persistent viruses may be reactivated under conditions of SARS-CoV-2-driven immunological dysregulation and infect new tissues, causing new symptoms. The Epstein–Barr virus (EBV) is a human gamma herpesvirus known to infect and generally become latent in more than 90% of the global population (91). The high rate of EBV infection in every region of the world is due to the spread of viruses from one host to another due to the lifelong persistence of EBV in the latent state and its recurrence in latently infected individuals (91). Several cohort studies have reported that reactivation of latent EBV under SARS-CoV-2-driven immune dysregulation is related to a higher prevalence of PASC symptoms (92–94). One study showed that EBV reactivation is associated with fatigue and neurocognitive dysfunction in patients with PASC (94). Immunological dysregulation involving the depletion of innate immune cells has been observed during the acute (95, 96) and convalescent phases (57, 58). Notably, we showed that the unrecovered depletion of DC could be attributed to persistent infection (17). Therefore, persistent SARS-CoV-2, which remains within the host, causes long-term immunological dysregulation and mediates the reactivation of other latent viruses, resulting in PASC symptoms. These clinical observations strongly support our proposal of potential contributors to PASC that are directly or indirectly caused by persistent infections.

As for possible measures for PASC, vaccination before infection can be one of the most effective methods for preventing the development of PASC (77–81, 97, 98). As shown in **Figures 6A and C**, the acute phase viral load is sufficiently reduced for at least one year after vaccination. Thus, the vaccination can suppress extensive tissue damage caused by a viral infection, thereby avoiding the potential contributors to PASC symptoms, especially concerned with the impact of unrepaired tissue injuries due to persistent viruses. Furthermore, booster vaccination is required at least once a year to maintain sufficient vaccination efficiency (**Figure 8**). These findings support that vaccinations adhering to the once-a-year schedule will be an efficient strategy in the management of PASC.

However, as mentioned above, the preventive effect of vaccine confers only partial protection against PASC symptoms; thus, additional mitigation strategies are necessary to reduce the long-term health consequences of SARS-CoV-2 infection. Vaccine therapy for patients with PASC is considered one of the treatments because of the high possibility of persistent infection behind the development of PASC. Among the patients experiencing PASC symptoms who had received vaccine therapy, 22–58% had improved, 18–31% had worsened, and 62–71% had remained unchanged (72, 74). To administer vaccine therapy in patients with PASC, it is necessary to make efforts to reduce the risk of exacerbation as much as possible. The improvement of PASC symptoms by vaccination that had been reported could be interpreted such that the potential contributors to PASC arising from persistent infection were partially eliminated or alleviated during the long-term but temporary reduction in the viral load (**Figure 5D**). However, the reason why several ten percent of patients with PASC had worsened could be attributed to the immediate rapid increase in viral load upon vaccination (**Figure 5D**). We examined how the combination with antiviral medication affected the viral load and found that the simultaneous ingestion of antiviral medication successfully suppressed the increase in viral load (**Figure 7C**). This finding implies that the combination of vaccination and antiviral medication is an effective and cost-efficient treatment for PASC.

### Limitations of the study

Infection with a conventional strain of SARS-CoV-2 was assumed in the baseline model; thus, 5.6 days was used as the time to symptom onset after infection (24). If we apply the mathematical model to a mutant strain, the parameters in the model must be recalibrated using the clinical data of infection with the mutant strain.

Generally, as the viral load decreases, the stochastic fluctuation effect on the time course of viral load becomes more pronounced, increasing the likelihood of the viral load transitioning to zero, especially in the vicinity of a minimum point in the viral load. However, since the virus-free equilibrium becomes an unstable state following infection, the temporal evolution by the mathematical model utilizing ordinary differential equations never reaches an endpoint even when the virus count drops below one in the host; instead, it tends towards a virus co-existence equilibrium. Nevertheless, since the virus coexistence equilibrium is in a stable state after infection, the possibility cannot be ruled out that not only patients who develop PASC but also asymptomatic patients who recover from the acute symptoms may develop persistent infection with some remaining within-host viruses.

Our model simulation predicted that, when the interval between vaccine doses exceeded two years, the antibody titer produced by a subsequent vaccination was similar to that observed after the initial dose of the primary vaccination series. Thus this level was markedly lower than the levels achieved after the second dose. To achieve the same level of antibody titer as the 2^nd^ dose of the primary vaccination series, continuing with the vaccine booster dose at least once a year is necessary. However, further clinical research is needed to confirm these findings in real-world settings.

Our findings imply that the use of antiviral drugs in combination with vaccines might serve as a beneficial treatment for PASC. This approach may help in lowering the risk of deterioration observed with vaccination alone, while also increasing the possibility of improvements in PASC. However, clinical trials that involve a large sample size and diverse patient populations are necessary to obtain robust evidence regarding the effectiveness of this combination therapy in treating PASC.

## Conclusions

We presented a mathematical model of the immune response to both SARS-CoV-2 infection and vaccination, with a focus on the development of PASC, also known as Long COVID. Our model incorporated immune memory cells, allowing us to explore the stability and kinetics of persistent infection and the impact of vaccination on PASC development. We provided insights into the dynamics of persistent infection and its potential contributions to PASC symptoms. Based on simulation experiments with the baseline model and models with varying parameters, we revealed the role of vaccination in preventing severe tissue damage caused by acute viral infection, which is one of key contributors to PASC development. We also analyzed the effectiveness of additional vaccine doses and their impact on antibody production, shedding light on the efficient timing of booster vaccinations. Our study proposes that the co-administration of antiviral medications with vaccination is a promising approach for treating PASC patients with persistent infection, where a temporal increase in viral load upon the vaccination can be suppressed by the antiviral medication.

## Methods

### Mathematical Model

In this study, based on cellular immunological knowledge, we developed a mathematical model comprising ordinary differential equations (ODEs) to investigate the host immune responses to SARS-CoV-2 infection and vaccination (Equations 1–23). In this model, the respiratory tract was assumed to be the initial site of infection (**Figure 2A**). However, SARS-CoV-2 can reach and infect cells in multiple organs and tissues via hematogenous diffusion from the heavily infected airways and lungs (12). Therefore, as proposed in our previous mathematical model (17), all cells expressing ACE2 were assumed to be the targets of SARS-CoV-2 infection. On the other hand, the vaccine was assumed to be administered to the shoulder musculature. Thus, the immune responses to antigens upon viral infection and vaccination occur at different sites. The most important difference in the immune response to viral infection and vaccination is that neither vaccine particles nor spike proteins can conduct self-replication unlike virus particles (**Figures 2A, B**). However, typical immune responses to vaccines are very similar to those of viral infections. Therefore, the case of viral infection will be explained below, and additional points related to vaccination will be supplemented where necessary.

The healthy cells were supplied at the rate ${\text{λ}}_{H}\;$ and underwent apoptosis at the rate $\delta_{H}$ (Equation 1). As in our previous model, it was assumed that uninfected cells were generated to return to a certain number, even if they were temporarily reduced due to viral infection. Thus, a model that satisfies this dynamic behavior under the balance between supply and death (Equation 1) was employed. The dendritic cells (Equation 3), naïve CD4^+^ T cells (Equation 11), naïve CD8^+^ T cells (Equation 13), and naïve B cells (Equation 18) were assumed to regenerate and die in the same manner.

The rate of infection of the healthy cells with free virus ( $\pi_{I}\left[H\right]\left[V\right])$ was divided by $\left\{1+\beta_{I}\left(\left[Iɡ\right]+\theta_{cross}\left[I{ɡ}^{vac}\right]\right)\left[V\right]\right\}$ (Equation 2a). Here, $\theta_{cross}$ was introduced as a reduction rate for cross reactions, e.g., between viral infection and vaccination, and assumed to be 0.8. Thus, in Equation 2a, infection was suppressed by the binding of Ig and Ig^vac^ produced by infection and vaccination, respectively, to the virus. Here, Ig was assumed to include antiviral antibodies acting against SARS-CoV-2 acquired upon seasonal human coronavirus infections as well as SARS-CoV-2 infections (**Supplementary Table S1** in the SM). This is because a cohort of SARS-CoV-2–uninfected individuals was found to possess antiviral antibodies against SARS-CoV-2 (99). In contrast to the viral infection, the rate of infection of the healthy cells with vaccine particles was simply given $\pi_{vac}\left[H\right]\left[Vac\right]$ (Equation 2b).

Dendritic cells that are recruited and activated by IFN1 (100) efficiently capture antibody-neutralized viruses via their Fc receptors (62–64) and transform into APC_R_ at infection sites. Therefore, the rate of DC transformation into APC_R_ was given as $\pi_{APC}\left[DC\right]\left[V\right]$ multiplied by $\left(1+\alpha_{recruit}\left[INF1\right]\right)\left\{1+\alpha_{APC}\left(\left[Iɡ\right]+\theta_{cross}\left[I{ɡ}^{vac}\right]\right)\right\}$ (Equation 4a). Viral replication is inhibited by IFN1-induced genes (101, 102). Hence, the viral replication rate was assumed to be proportional to the inverse of $\left(1+\beta_{V}\left[INF1\right]\right)$ (Equation 5a). In Equation 5a, the virus neutralized by antibodies was removed at a rate proportional to $\gamma_{Iɡ}\left(\left[Iɡ\right]+\theta_{cross}\left[I{ɡ}^{vac}\right]\right)$ . The production rates of IFN1 by I and APC_R_ were $\sigma_{I}\left[I\right]$ and $\sigma_{APC}\left[APC_{R}\right]$ , respectively (Equation 7a), and $\sigma_{I}$ was assumed to be 1,000-fold lower than $\sigma_{APC}$ (**Supplementary Table S2** in the SM) due to the several mechanisms employed by SARS-CoV-2 to evade the IFN1-mediated immune response (102). The rate of production of spike protein (Equation 5b) was assumed to have a form similar to that of viral replication (Equation 5a). In addition, in the same manner as in Equation 5a, the spike protein bound by antibodies was removed at a rate proportional to $\gamma_{Iɡ}^{vac}\left(\left[I{ɡ}^{vac}\right]+\theta_{cross}\left[Iɡ\right]\right)$ (Equation 5b). In the present study, chemokine receptor ligand (CXCL) that was produced by infected cells (103) (Equation 8a) was newly introduced and assumed to efficiently recruit memory B cells (104) and memory CTL (105) as well as CTL (106) to sites of infection. The administration of the vaccines was modeled by Equation 9 using **Supplementary Equation S1** in the SM.

APC_R_ was assumed to migrate into lymph nodes with the rate $\mu_{APC}\left[APC_{R}\right]$ (Equation 10a). The development of naïve CD4^+^T_0_ cells into Th1 and Tfh cells by APC_L_ (107) is stimulated by IFN1 (108, 109). Therefore, the rates of CD4^+^T_0_ transformation into Th1 and Tfh cells, $\pi_{Th1}\left[APC_{L}\right]\left[CD4^{+}T_{o}\right]$ (Equation 12a) and $\pi_{Tfh}\left[APC_{L}\right]\left[CD4^{+}T_{o}\right]$ (Equation 17a), were assumed to be multiplied by $\left(1+\alpha_{Th1}\left[INF1\right]\right)$ and $\left(1+\alpha_{Tfh}\left[INF1\right]\right)$ , respectively. APC_L_ and Th1 cells activate CD8^+^ T_0_ cells, which then differentiate into CTL_L_ and memory CTL_L_ cells (107). Thus, the rates of CD8^+^ T_0_ transformation into CTL_L_, and mCTL_L_ cells were calculated as $\left[APC_{L}\right]\left[Th1\right]\left[CD8^{+}T_{o}\right]$ multiplied by $\pi_{CTL}$ (Equation 14a) and $\pi_{mCTL}$ (Equation 15a), respectively. CTL_L_ is activated by IFN1 (108) and recruited toward the sites of infection by CXCL (106); therefore, the migration rate of CTL_L_ was assumed to be $\mu_{CTL}\left(1+\alpha_{recruit}\left[INF1\right]\right)\left(1+\omega_{recruit}\left[CXCL\right]\right)\left[CTL_{L}\right]$ (Equation 6a). mCTLL is recruited by CXCL and resides in the peripheral tissue sites of pathogen encounters, working as mCTL_R_ (105). mCTL_R_ was assumed to go back to lymph nodes with the rate $\mu_{mCTLL}\left[mCTL_{R}\right]$ and circulate (Equation 16a). CTL_R_ directly kills infected cells at a rate of $k_{I\_CTL}\left[I\right]\left[CTL_{R}\right]$ (Equation 2a). However, mCTL_R_ that resides in peripheral tissue sites requires APC_R_ for reactivation (68); therefore, the rate of killing infected cells was assumed to be $k_{I\_mCTL}\left[I\right]\left[V\right]\left[APC_{R}\right]\left[mCTL_{R}\right]$ (Equation 2a).

APC_L_ and Tfh cells activate naïve B_0_ cells, which differentiate into pBS, pBL, and memory B cells (67). Thus, the rates of B_0_ transformation into pBS, pBL, and memory B cells were assumed to be $\left[APC_{L}\right]\left[Tfh\right]\left[B_{o}\right]$ multiplied by $\pi_{pBS}$ (Equation 19a), $\pi_{pBL}$ (Equation 20a), and $\pi_{mBL}$ (Equation 22a), respectively. mB_L_ cells are recruited by CXCL toward the sites of infection (104); therefore, the migration rate was assumed to be $\mu_{mBR}\left(1+\omega_{recruit}\left[CXCL\right]\right)\left[mB_{L}\right]$ (Equation 23a). mB_R_ cells reside in the infected regions in anticipation of reinfection (104), whereas parts of them were assumed to go back to lymph nodes with the rate $\mu_{mBL}\left[mB_{R}\right]$ and circulate (Equation 23a). mB_R_ cells recall upon recognizing antigens via receptors for the specific antigens (66), thus were assumed to transform into pBL cells (67) with the rate $\pi_{mB\_pBL}\left[V\right]\left[mB_{R}\right]$ (Equation 20a). pBS and pBL cells produced Ig with the rates of $\pi_{IɡS}\left[pBS\right]$ and $\pi_{IɡL}\left[pBL\right]$ , respectively, and the Ig degradation rate was given by $\delta_{Iɡ}\left[Ig\right]$ (Equation 21a). In Equation 21a, Ig was consumed upon binding to virus with the rate $\xi_{Iɡ}\left[Iɡ\right]\left[V\right]$ and upon binding to spike protein with the rate $\gamma_{Iɡ}^{vac}\theta_{cross}\left[Iɡ\right]\left[S\right]$ . In addition, to examine the immune responses when viral infection and vaccination occur in parallel, cross-interactions between viral infection and vaccination, as schematically depicted in **Figures 2C, D**, were incorporated into the model. All the ODEs in the model are listed below.

Sites of infection/vaccination


(1) $$\begin{array}{c} d\left[H\right]/dt={\text{λ}}_{H}-\delta_{H}\left[H\right]\\ -\pi_{I}\left[H\right]\left[V\right]/\left\{1+\beta_{I}\left(\left[Iɡ\right]+\theta_{cross}\left[I{ɡ}^{vac}\right]\right)\left[V\right]\right\}\\ -\pi_{vac}\left[H\right]\left[Vac\right] \end{array}$$

(2a) $$\begin{array}{c} d\left[I\right]/dt=\pi_{I}\left[H\right]\left[V\right]/\left\{1+\beta_{I}\left(\left[Iɡ\right]+\theta_{cross}\left[I{ɡ}^{vac}\right]\right)\left[V\right]\right\}-\delta_{I}\left[I\right]\\ -k_{I\_CTL}\left[I\right]\left[CTL_{R}\right]-\theta_{cross}k_{I\_CTL}^{vac}\left[I\right]\left[CTL_{R}^{vac}\left(c\right)\right]\\ -k_{I\_mCTL}\left[I\right]\left[V\right]\left[APC_{R}\right]\left[mCTL_{R}\right]\\ -\theta_{cross}k_{I\_mCTL}\left[I\right]\left[V\right]\left[APC_{R}\right]\left[mCTL_{R}^{vac}\left(c\right)\right] \end{array}$$

(2b) $$\begin{array}{c} d\left[H^{vac}\right]/dt=\pi_{vac}\left[H\right]\left[Vac\right]-\delta_{I}^{vac}\left[H^{vac}\right]\\ -k_{I\_CTL}^{vac}\left[H^{vac}\right]\left[CTL_{R}^{vac}\right]\\ -\theta_{cross}k_{I\_CTL}\left[H^{vac}\right]\left[CTL_{R}\left(c\right)\right]\\ -k_{I\_mCTL}^{vac}\left[H^{vac}\right]\left[S\right]\left[APC_{R}^{vac}\right]\left[mCTL_{R}^{vac}\right]\\ -\theta_{cross}k_{I\_mCTL}^{vac}\left[H^{vac}\right]\left[S\right]\left[APC_{R}^{vac}\right]\left[mCTL_{R}\left(c\right)\right] \end{array}$$

(3) $$\begin{array}{c} d\left[DC\right]/dt={\text{λ}}_{DC}-\delta_{DC}\left[DC\right]-\pi_{APC}(1\\ +\alpha_{recruit}\left[INF1\right])\{1+\alpha_{APC}\left(\left[Iɡ\right]+\theta_{cross}\left[I{ɡ}^{vac}\right]\right)\}\left[DC\right]\left[V\right]\\ -\pi_{APC}^{vac}(1\\ +\alpha_{recruit}\left[INF1^{vac}\right])\left\{1+\alpha_{APC}^{vac}\left(\left[I{ɡ}^{vac}\right]+\theta_{cross}\left[Iɡ\right]\right)\right\}\left[DC\right]\left[S\right] \end{array}$$

(4a) $$\begin{array}{c} d\left[APC_{R}\right]/dt=\pi_{APC}(1\\ +\alpha_{recruit}\left[INF1\right])\left\{1+\alpha_{APC}\left(\left[Iɡ\right]+\theta_{cross}\left[I{ɡ}^{vac}\right]\right)\right\}\left[DC\right]\left[V\right]\\ -\delta_{APC_{R}}\left[APC_{R}\right]-\mu_{APC}\left[APC_{R}\right] \end{array}$$

(4b) $$\begin{array}{c} d\left[APC_{R}^{vac}\right]/dt=\pi_{APC}^{vac}(1\\ +\alpha_{recruit}\left[INF1^{vac}\right])\{1+\alpha_{APC}^{vac}\left(\left[I{ɡ}^{vac}\right]+\theta_{cross}\left[Iɡ\right]\right)\}\left[DC\right]\left[S\right]\\ -\delta_{APC_{R}}^{vac}\left[APC_{R}^{vac}\right]-\mu_{APC}^{vac}\left[APC_{R}^{vac}\right] \end{array}$$

(5a) $$\begin{array}{c} d\left[V\right]/dt=\pi_{V}\left[I\right]/\left(1+\beta_{V}\left[INF1\right]\right)-\delta_{V}\left[V\right]\\ -\pi_{I}\left[H\right]\left[V\right]/\{1+\beta_{I}\left(\left[Iɡ\right]+\theta_{cross}\left[I{ɡ}^{vac}\right]\right)\left[V\right]\}\\ -\pi_{APC}(1\\ +\alpha_{recruit}\left[INF1\right])\left\{1+\alpha_{APC}\left(\left[Iɡ\right]+\theta_{cross}\left[I{ɡ}^{vac}\right]\right)\right\}\left[DC\right]\left[V\right]\\ -\gamma_{Iɡ}\left(\left[Iɡ\right]+\theta_{cross}\left[I{ɡ}^{vac}\right]\right)\left[V\right] \end{array}$$

(5b) $$\begin{array}{c} d\left[S\right]/dt=\pi_{s}\left[H^{vac}\right]/\left(1+\beta_{S}\left[INF1^{vac}\right]\right)-\delta_{S}\left[S\right]-\pi_{APC}^{vac}(1\\ +\alpha_{recruit}\left[INF1^{vac}\right])\left\{1+\alpha_{APC}^{vac}\left(\left[I{ɡ}^{vac}\right]+\theta_{cross}\left[Iɡ\right]\right)\right\}\left[DC\right]\left[S\right]\\ -\gamma_{Iɡ}^{vac}\left(\left[I{ɡ}^{vac}\right]+\theta_{cross}\left[Iɡ\right]\right)\left[S\right] \end{array}$$

(6a) $$\begin{array}{c} d\left[CTL_{R}\right]/dt=\mu_{CTL}\left(1+\alpha_{recruit}\left[INF1\right]\right)(1\\ +\omega_{recruit}\left[CXCL\right])\left[CTL_{L}\right]-\delta_{CTL}\left[CTL_{R}\right] \end{array}$$

(6b) $$\begin{array}{c} d\left[CTL_{R}^{vac}\right]/dt=\mu_{CTL}^{vac}\left(1+\alpha_{recruit}\left[INF1^{vac}\right]\right)(1\\ +\omega_{recruit}\left[CXCL^{vac}\right])\left[CTL_{L}^{vac}\right]-\delta_{CTL}^{vac}\left[CTL_{R}^{vac}\right] \end{array}$$

(6c) $$\begin{array}{c} d\left[CTL_{R}\left(c\right)\right]/dt=\mu_{CTL}(1\\ +\alpha_{recruit}\left[INF1^{vac}\right])\omega_{recruit}\left[CXCL^{vac}\right]\left[CTL_{L}\right]\\ -\delta_{CTL}\left[CTL_{R}\left(c\right)\right] \end{array}$$

(6d) $$\begin{array}{c} d\left[CTL_{R}^{vac}\left(c\right)\right]/dt\\ =\mu_{CTL}^{vac}\left(1+\alpha_{recruit}\left[INF1\right]\right)\omega_{recruit}\left[CXCL\right]\left[CTL_{L}^{vac}\right]\\ -\delta_{CTL}^{vac}\left[CTL_{R}^{vac}\left(c\right)\right] \end{array}$$

(7a) $$d\left[IFN1\right]/dt=\sigma_{I}\left[I\right]+\sigma_{APC}\left[APC_{R}\right]-\delta_{IFN1}\left[IFN1\right]$$

(7b) $$d\left[INF1^{vac}\right]/dt=\sigma_{I}^{vac}\left[H^{vac}\right]+\sigma_{APC}^{vac}\left[APC_{R}^{vac}\right]-\delta_{IFN1}\left[INF1^{vac}\right]$$

(8a) $$d\left[CXCL\right]/dt=\sigma_{CXCL}\left[I\right]-\delta_{CXCL}\left[CXCL\right]$$

(8b) $$d\left[CXCL^{vac}\right]/dt=\sigma_{CXCL}^{vac}\left[H^{vac}\right]-\delta_{CXCL}\left[CXCL^{vac}\right]$$

(9) $$d\left[Vac\right]/dt=\sum_{i}J_{i}^{vac}\left(t-t_{i}^{vac}\right)-\delta_{vac}\left[Vac\right]-\pi_{vac}\left[H\right][Vac].$$

Differentiation of naïve CD8^+^ T cells into CTLs in lymph nodes


(10a) $$d\left[APC_{L}\right]/dt=\mu_{APC}\left[APC_{R}\right]-\delta_{APC_{L}}\left[APC_{L}\right]$$

(10b) $$d\left[APC_{L}^{vac}\right]/dt=\mu_{APC}^{vac}\left[APC_{R}^{vac}\right]-\delta_{APC_{L}}^{vac}\left[APC_{L}^{vac}\right]$$

(11) $$\begin{array}{c} d\left[CD4^{+}T_{o}\right]/dt={\text{λ}}_{CD4}-\delta_{CD4}\left[CD4^{+}T_{o}\right]-\pi_{Th1}(1\\ +\alpha_{Th1}\left[INF1\right])\left[APC_{L}\right]\left[CD4^{+}T_{o}\right]-\pi_{Tfh}(1\\ +\alpha_{Tfh}\left[INF1\right])\left[APC_{L}\right]\left[CD4^{+}T_{o}\right]-\pi_{Th1}^{vac}(1\\ +\alpha_{Th1}^{vac}\left[INF1^{vac}\right])\left[APC_{L}^{vac}\right]\left[CD4^{+}T_{o}\right]-\pi_{Tfh}^{vac}(1\\ +\alpha_{Tfh}^{vac}\left[INF1^{vac}\right])\left[APC_{L}^{vac}\right]\left[CD4^{+}T_{o}\right] \end{array}$$

(12a) $$\begin{array}{c} d\left[Th1\right]/dt=\pi_{Th1}\left(1+\alpha_{Th1}\left[INF1\right]\right)\left[APC_{L}\right]\left[CD4^{+}T_{o}\right]\\ -\delta_{Th1}\left[Th1\right] \end{array}$$

(12b) $$\begin{array}{c} d\left[Th1^{vac}\right]/dt=\pi_{Th1}^{vac}\left(1+\alpha_{Th1}^{vac}\left[INF1^{vac}\right]\right)\left[APC_{L}^{vac}\right]\left[CD4^{+}T_{o}\right]\\ -\delta_{Th1}^{vac}\left[Th1^{vac}\right] \end{array}$$

(13) $$\begin{array}{c} d\left[CD8^{+}T_{o}\right]/dt={\text{λ}}_{CD8}-\delta_{CD8}\left[CD8^{+}T_{o}\right]-(\pi_{CTL}\\ +\pi_{mCTL})\left[APC_{L}\right]\left[Th1\right]\left[CD8^{+}T_{o}\right]-(\pi_{CTL}^{vac}\\ +\pi_{mCTL}^{vac})\left[APC_{L}^{vac}\right]\left[Th1^{vac}\right]\left[CD8^{+}T_{o}\right] \end{array}$$

(14a) $$\begin{array}{c} d\left[CTL_{L}\right]/dt=\pi_{CTL}\left[APC_{L}\right]\left[Th1\right]\left[CD8^{+}T_{o}\right]-\delta_{CTL}\left[CTL_{L}\right]\\ -\mu_{CTL}(1+\alpha_{recruit}\left[INF1\right])(1\\ +\omega_{recruit}\left[CXCL\right])\left[CTL_{L}\right]-\mu_{CTL}(1\\ +\alpha_{recruit}\left[INF1^{vac}\right])\omega_{recruit}\left[CXCL^{vac}\right]\left[CTL_{L}\right] \end{array}$$

(14b) $$\begin{array}{c} d\left[CTL_{L}^{vac}\right]/dt=\pi_{CTL}^{vac}\left[APC_{L}^{vac}\right]\left[Th1^{vac}\right]\left[CD8^{+}T_{o}\right]\\ -\delta_{CTL}^{vac}\left[CTL_{L}^{vac}\right]-\mu_{CTL}^{vac}(1\\ +\alpha_{recruit}\left[INF1^{vac}\right])(1\\ +\omega_{recruit}\left[CXCL^{vac}\right])\left[CTL_{L}^{vac}\right]-\mu_{CTL}^{vac}(1\\ +\alpha_{recruit}\left[INF1\right])\omega_{recruit}\left[CXCL\right]\left[CTL_{L}^{vac}\right] \end{array}$$

CD8^+^ memory T cell generation


(15a) $$\begin{array}{c} d\left[mCTL_{L}\right]/dt=\pi_{mCTL}\left[APC_{L}\right]\left[Th1\right]\left[CD8^{+}T_{o}\right]\\ +\mu_{mCTLL}\left(\left[mCTL_{R}\right]+\left[mCTL_{R}\left(c\right)\right]\right)\\ -\mu_{mCTLR}\left(1+\omega_{recruit}\left[CXCL\right]\right)\left[mCTL_{L}\right]\\ -\mu_{mCTLR}\omega_{recruit}\left[CXCL^{vac}\right]\left[mCTL_{L}\right]\\ -\delta_{mCTL}\left[mCTL_{L}\right] \end{array}$$

(15b) $$\begin{array}{c} d\left[mCTL_{L}^{vac}\right]/dt\\ =\pi_{mCTL}^{vac}\left[APC_{L}^{vac}\right]\left[Th1^{vac}\right]\left[CD8^{+}T_{o}\right]+\mu_{mCTLL}^{vac}(\left[mCTL_{R}^{vac}\right]\\ +\left[mCTL_{R}^{vac}\left(c\right)\right])-\mu_{mCTLR}^{vac}(1\\ +\omega_{recruit}\left[CXCL^{vac}\right])\left[mCTL_{L}^{vac}\right]\\ -\mu_{mCTLR}^{vac}\omega_{recruit}\left[CXCL\right]\left[mCTL_{L}^{vac}\right]-\delta_{mCTL}^{vac}\left[mCTL_{L}^{vac}\right] \end{array}$$

(16a) $$\begin{array}{c} d\left[mCTL_{R}\right]/dt=\mu_{mCTLR}\left(1+\omega_{recruit}\left[CXCL\right]\right)\left[mCTL_{L}\right]\\ -\mu_{mCTLL}\left[mCTL_{R}\right]-\delta_{mCTL}\left[mCTL_{R}\right] \end{array}$$

(16b) $$\begin{array}{c} d\left[mCTL_{R}^{vac}\right]/dt\\ =\mu_{mCTLR}^{vac}\left(1+\omega_{recruit}\left[CXCL^{vac}\right]\right)\left[mCTL_{L}^{vac}\right]\\ -\mu_{mCTLL}^{vac}\left[mCTL_{R}^{vac}\right]-\delta_{mCTL}^{vac}\left[mCTL_{R}^{vac}\right] \end{array}$$

(16c) $$\begin{array}{c} d\left[mCTL_{R}\left(c\right)\right]/dt\\ =\mu_{mCTLR}\omega_{recruit}\left[CXCL^{vac}\right]\left[mCTL_{L}\right]-\mu_{mCTLL}\left[mCTL_{R}\left(c\right)\right]\\ -\delta_{mCTL}\left[mCTL_{R}\left(c\right)\right] \end{array}$$

(16d) $$\begin{array}{c} d\left[mCTL_{R}^{vac}\left(c\right)\right]/dt\\ =\mu_{mCTLR}^{vac}\omega_{recruit}\left[CXCL\right]\left[mCTL_{L}^{vac}\right]-\mu_{mCTLL}^{vac}\left[mCTL_{R}^{vac}\left(c\right)\right]\\ -\delta_{mCTL}^{vac}\left[mCTL_{R}^{vac}\left(c\right)\right] \end{array}$$

Ig production by pBS and pBL


(17a) $$\begin{array}{c} d\left[Tfh\right]/dt=\pi_{Tfh}\left(1+\alpha_{Tfh}\left[INF1\right]\right)\left[APC_{L}\right]\left[CD4^{+}T_{o}\right]\\ -\delta_{Tfh}\left[Tfh\right] \end{array}$$

(17b) $$\begin{array}{c} d\left[Tfh^{vac}\right]/dt=\pi_{Tfh}^{vac}\left(1+\alpha_{Tfh}^{vac}\left[INF1^{vac}\right]\right)\left[APC_{L}^{vac}\right]\left[CD4^{+}T_{o}\right]\\ -\delta_{Tfh}^{vac}\left[Tfh^{vac}\right] \end{array}$$

(18) $$\begin{array}{c} d\left[B_{o}\right]/dt={\text{λ}}_{B}-\delta_{B}\left[B_{o}\right]-(\pi_{pBS}+\pi_{pBL}\\ +\pi_{mBL})\left[APC_{L}\right]\left[Tfh\right]\left[B_{o}\right]-(\pi_{pBS}^{vac}+\pi_{pBL}^{vac}\\ +\pi_{mBL}^{vac})\left[APC_{L}^{vac}\right]\left[Tfh^{vac}\right]\left[B_{o}\right] \end{array}$$

(19a) $$d\left[pBS\right]/dt=\pi_{pBS}\left[APC_{L}\right]\left[Tfh\right]\left[B_{o}\right]-\delta_{pBS}\left[pBS\right]$$

(19b) $$d\left[pBS^{vac}\right]/dt=\pi_{pBS}^{vac}\left[APC_{L}^{vac}\right]\left[Tfh^{vac}\right]\left[B_{o}\right]-\delta_{pBS}^{vac}\left[pBS^{vac}\right]$$

(20a) $$\begin{array}{c} d\left[pBL\right]/dt=\pi_{pBL}\left[APC_{L}\right]\left[Tfh\right]\left[B_{o}\right]+\pi_{mB\_pBL}\left[V\right]\left[mB_{R}\right]\\ +\theta_{cross}\pi_{mB\_pBL}^{vac}\left[S\right]\left[mB_{R}\left(c\right)\right]-\delta_{pBL}\left[pBL\right] \end{array}$$

(20b) $$\begin{array}{c} d\left[pBL^{vac}\right]/dt=\pi_{pBL}^{vac}\left[APC_{L}^{vac}\right]\left[Tfh^{vac}\right]\left[B_{o}\right]\\ +\pi_{mB\_pBL}^{vac}\left[S\right]\left[mB_{R}^{vac}\right]\\ +\theta_{cross}\pi_{mB\_pBL}\left[V\right]\left[mB_{R}^{vac}\left(c\right)\right]-\delta_{pBL}^{vac}\left[pBL^{vac}\right] \end{array}$$

(21a) $$\begin{array}{c} d\left[Iɡ\right]/dt=\pi_{IɡS}\left[pBS\right]+\pi_{IɡL}\left[pBL\right]-\delta_{Iɡ}\left[Iɡ\right]-\xi_{Iɡ}\left[Iɡ\right]\left[V\right]\\ -\gamma_{Iɡ}^{vac}\theta_{cross}\left[Iɡ\right]\left[S\right] \end{array}$$

(21b) $$\begin{array}{c} d\left[I{ɡ}^{vac}\right]/dt=\pi_{IɡS}^{vac}\left[pBS^{vac}\right]+\pi_{IɡL}^{vac}\left[pBL^{vac}\right]-\delta_{Iɡ}^{vac}\left[I{ɡ}^{vac}\right]\\ -\xi_{Iɡ}\theta_{cross}\left[I{ɡ}^{vac}\right]\left[V\right]-\gamma_{Iɡ}^{vac}\left[I{ɡ}^{vac}\right]\left[S\right] \end{array}$$

Memory B cell generation


(22a) $$\begin{array}{c} d\left[mB_{L}\right]/dt=\pi_{mBL}\left[APC_{L}\right]\left[Tfh\right]\left[B_{o}\right]+\mu_{mBL}(\left[mB_{R}\right]\\ +\left[mB_{R}\left(c\right)\right])-\mu_{mBR}\left(1+\omega_{recruit}\left[CXCL\right]\right)\left[mB_{L}\right]\\ -\mu_{mBR}\omega_{recruit}\left[CXCL^{vac}\right]\left[mB_{L}\right]-\delta_{mBL}\left[mB_{L}\right] \end{array}$$

(22b) $$\begin{array}{c} d\left[mB_{L}^{vac}\right]/dt=\pi_{mBL}^{vac}\left[APC_{L}^{vac}\right]\left[Tfh^{vac}\right]\left[B_{o}\right]+\mu_{mBL}^{vac}(\left[mB_{R}^{vac}\right]\\ +\left[mB_{R}^{vac}\left(c\right)\right])-\mu_{mBR}^{vac}(1\\ +\omega_{recruit}\left[CXCL^{vac}\right])\left[mB_{L}^{vac}\right]\\ -\mu_{mBR}^{vac}\omega_{recruit}\left[CXCL\right]\left[mB_{L}^{vac}\right]-\delta_{mBL}^{vac}\left[mB_{L}^{vac}\right] \end{array}$$

(23a) $$\begin{array}{c} d\left[mB_{R}\right]/dt=\mu_{mBR}\left(1+\omega_{recruit}\left[CXCL\right]\right)\left[mB_{L}\right]-\mu_{mBL}\left[mB_{R}\right]\\ -\pi_{mB\_pBL}\left[mB_{R}\right]\left[V\right]-\delta_{mBR}\left[mB_{R}\right] \end{array}$$

(23b) $$\begin{array}{c} d\left[mB_{R}^{vac}\right]/dt=\mu_{mBR}^{vac}\left(1+\omega_{recruit}\left[CXCL^{vac}\right]\right)\left[mB_{L}^{vac}\right]\\ -\mu_{mBL}^{vac}\left[mB_{R}^{vac}\right]-\pi_{mB\_pBL}^{vac}\left[mB_{R}^{vac}\right]\left[S\right]\\ -\delta_{mBR}^{vac}\left[mB_{R}^{vac}\right] \end{array}$$

(23c) $$\begin{array}{c} d\left[mB_{R}\left(c\right)\right]/dt=\mu_{mBR}\omega_{recruit}\left[CXCL^{vac}\right]\left[mB_{L}\right]-\mu_{mBL}\left[mB_{R}\left(c\right)\right]\\ -\theta_{cross}\pi_{mB\_pBL}^{vac}\left[S\right]\left[mB_{R}\left(c\right)\right]-\delta_{mBR}\left[mB_{R}\left(c\right)\right] \end{array}$$

(23d) $$\begin{array}{c} d\left[mB_{R}^{vac}\left(c\right)\right]/dt=\mu_{mBR}^{vac}\omega_{recruit}\left[CXCL\right]\left[mB_{L}^{vac}\right]\\ -\mu_{mBL}^{vac}\left[mB_{R}^{vac}\left(c\right)\right]\\ -\theta_{cross}\pi_{mB\_pBL}\left[V\right]\left[mB_{R}^{vac}\left(c\right)\right]\\ -\delta_{mBR}^{vac}\left[mB_{R}^{vac}\left(c\right)\right] \end{array}$$

3C-like protease inhibitor

Equation 5ab is used instead of Equation 5a and Equation 24 is added, when 3CLPI is administrated.


(5ab) $$\begin{array}{c} d\left[V\right]/dt=\pi_{V}\left[I\right]/\left(1+\beta_{V}\left[INF1\right]+\beta_{3C}\left[3CLPI\right]\right)-\delta_{V}\left[V\right]\\ -\pi_{I}\left[H\right]\left[V\right]/\left\{1+\beta_{I}\left(\left[Iɡ\right]+\theta_{cross}\left[I{ɡ}^{vac}\right]\right)\left[V\right]\right\}\\ -\pi_{APC}(1\\ +\alpha_{recruit}\left[INF1\right])\left\{1+\alpha_{APC}\left(\left[Iɡ\right]+\theta_{cross}\left[I{ɡ}^{vac}\right]\right)\right\}\left[DC\right]\left[V\right]\\ -\gamma_{Ig}\left(\left[Iɡ\right]+\theta_{cross}\left[I{ɡ}^{vac}\right]\right)\left[V\right] \end{array}$$

(24) $$d\left[3CLPI\right]/dt=J^{3CLPI}-\delta_{3CLPI}\left[3CLPI\right]$$

The explanation of each equation described above is summarized in **Table 1**.

**Table 1:** Explanation of the terms included on the right-hand side of each equation.

Eqn. No.	Explanation of the terms on the right-hand side.
1	The regeneration of healthy cell [H] , natural death of [H] , and decrease in [H] by viral and vaccine infection.
2a	The increase in infected cell [I] by viral infections, natural death of [I] , and killing of [I] by various cytotoxic T lymphocytes (CTL) including the memory CTL.
2b	The increase in infected cell $\left[{\text{H}}^{\text{vac}}\right]$ by vaccine infection, natural death of $\left[{\text{H}}^{\text{vac}}\right]$ , and killing of $\left[{\text{H}}^{\text{vac}}\right]$ by various CTLs including the memory CTLs.
3	The regeneration and natural death of dendritic cell [DC] , decrease in [DC] upon transformation into antigen-presenting cells (APC) for virus and vaccine.
4a	The increase in APC at sites of infection $\left[\text{AP}{\text{C}}_{\text{R}}\right]$ upon up taking viruses, natural death of $\left[\text{AP}{\text{C}}_{\text{R}}\right]$ , and decrease in $\left[\text{AP}{\text{C}}_{\text{R}}\right]$ due to the migration into lymph nodes.
4b	The increase in APC at sites of vaccine administration $\left[{\text{AP}}_{C\text{R}}^{\text{vac}}\right]$ upon up taking spike protein antigens, natural death of $\left[{\text{AP}}_{C\text{R}}^{\text{vac}}\right]$ , and decrease in $\left[{\text{AP}}_{C\text{R}}^{\text{vac}}\right]$ due to the migration into lymph nodes.
5a	The virus replication by infected cells, natural death of viruses, decrease in [V] upon the viral infection to healthy cells, phagocytosis of [V] by dendritic cells, and neutralization of [V] by antibodies.
5b	The production of spike protein antigens [S] by vaccine-infected cells $\left[{\text{H}}^{\text{vac}}\right]$ , natural degradation of [S] , phagocytosis of [S] by dendritic cells, and neutralization of [V] by antibodies.
6a	The increase in virus-specific CTL at sites of infection $\left[\text{CT}L_{\text{R}}\right]$ upon migration of $\left[\text{CT}L_{\text{L}}\right]$ from lymph nodes and natural death of $\left[\text{CT}L_{\text{R}}\right]$ .
6b	The increase in vaccine-mediated CTL at sites of vaccine administration $\left[{\text{CTL}}_{\text{R}}^{\text{vac}}\right]$ upon migration of $\left[{\text{CTL}}_{\text{L}}^{\text{vac}}\right]$ from lymph nodes and natural death of $\left[{\text{CTL}}_{\text{R}}^{\text{vac}}\right]$ .
6c	The increase in virus-specific CTL at sites of vaccine administration $\left[\text{CT}{\text{L}}_{\text{R}}\left(\text{c}\right)\right]$ upon migration of $\left[\text{CT}L_{\text{L}}\right]$ from lymph nodes and natural death of $\left[\text{CT}L_{\text{R}}\left(\text{c}\right)\right]$ .
6d	The increase in vaccine-mediated CTL at sites of infection $\left[{\text{CTL}}_{\text{R}}^{\text{vac}}\left(\text{c}\right)\right]$ upon migration of $\left[{\text{CTL}}_{\text{L}}^{\text{vac}}\right]$ from lymph nodes and natural death of $\left[{\text{CTL}}_{\text{R}}^{\text{vac}}\left(\text{c}\right)\right]$ .
7a	The increase in type-I interferon [INF1] produced by [I] and $\left[\text{AP}C_{\text{R}}\right]$ and natural degradation of [INF1] .
7b	The increase in type-I interferon $\left[\text{INF}1^{\text{vac}}\right]$ produced by $\left[{\text{H}}^{\text{vac}}\right]$ and $\left[{\text{APC}}_{\text{R}}^{\text{vac}}\right]$ and natural degradation of $\left[\text{INF}1^{\text{vac}}\right]$ .
8a	The increase in CXC chemokine receptor ligand (CXCL) [CXCL] produced by [I] and natural degradation of [CXCL] .
8b	The increase in vaccine-mediated CXCL $\left[\text{CXC}L^{\text{vac}}\right]$ produced by $\left[{\text{H}}^{\text{vac}}\right]$ and natural degradation of $\left[\text{CXC}L^{\text{vac}}\right]$ .
9	The influx of vaccine particle [Vac] , natural degradation of [Vac] , and decrease in [Vac] upon up taking of [Vac] by [H] .
10a	The increase in $\left[\text{AP}{\text{C}}_{\text{l}}\right]$ at lymph nodes upon migration of $\left[\text{AP}{\text{C}}_{\text{R}}\right]$ from sites of infection and natural death of $\left[\text{AP}{\text{C}}_{\text{L}}\right]$ .
10b	The increase in $\left[{\text{AP}}_{C\text{L}}^{\text{vac}}\right]$ at lymph nodes upon migration of $\left[{\text{AP}}_{C\text{R}}^{\text{vac}}\right]$ from sites of vaccine administration and natural death of $\left[{\text{AP}}_{C\text{L}}^{\text{vac}}\right]$ .
11	The regeneration and natural death of naïve CD4^+^ T cell $\left[\text{CDD}4^{+}{\text{T}}_{\text{o}}\right]$ and decrease in $\left[\text{CD}4^{+}{\text{T}}_{\text{o}}\right]$ upon transformation into virus-specific type I helper T cells [TH1] , virus-specific follicular helper T cells [Tfh] , vaccine-mediated type I helper T cells $\left[\text{TH}1^{\text{vac}}\right]$ , and vaccine-mediated follicular helper T cells $\left[\text{Tf}{\text{h}}^{\text{vac}}\right]$ .
12a	The increase in [Th1] upon transformation of $\left[\text{CD}4^{+}{\text{T}}_{\text{o}}\right]$ and natural death of [Th1] .
12b	The increase in $\left[\text{Th}1^{\text{vac}}\right]$ upon transformation of $\left[\text{CD}4^{+}{\text{T}}_{\text{o}}\right]$ and natural death of $\left[\text{Th}1^{\text{vac}}\right]$ .
13	The regeneration and natural death of naïve CD8^+^ T cells $\left[\text{CD}8^{+}{\text{T}}_{\text{o}}\right]$ and decrease in $\left[\text{CD}8^{+}{\text{T}}_{o}\right]$ upon transformation into virus-specific cytotoxic T lymphocytes $\left[\text{CT}{\text{L}}_{\text{L}}\right]$ and vaccine-mediated cytotoxic T lymphocytes $\left[{\text{CTL}}_{\text{L}}^{\text{vac}}\right]$ .
14a	The increase in $\left[\text{CT}L_{\text{R}}\right]$ upon transformation of $\left[\text{CDD}8^{+}{\text{T}}_{o}\right]$ , natural death, and decrease in $\left[\text{CT}L_{\text{L}}\right]$ with the migration of $\left[\text{CT}L_{\text{L}}\right]$ toward sites of infection and vaccine administration.
14b	The increase in $\left[{\text{CTL}}_{\text{L}}^{\text{vac}}\right]$ upon transformation of $\left[\text{CD}8^{+}{\text{T}}_{o}\right]$ , natural death, and decrease in $\left[{\text{CTL}}_{\text{L}}^{\text{vac}}\right]$ with the migration of $\left[{\text{CTL}}_{\text{L}}^{\text{vac}}\right]$ toward sites of vaccine administration and infection.
15a	The increase in virus-specific cytotoxic memory T cells at lymph nodes $\left[\text{mCT}L_{\text{L}}\right]$ upon transformation of $\left[\text{CD}8^{+}{\text{T}}_{o}\right]$ , natural death of $\left[\text{mCT}L_{\text{L}}\right]$ , increase in $\left[\text{mCT}L_{\text{L}}\right]$ with migration of virus-specific cytotoxic memory T cells at sites of infection $\left[\text{mCT}L_{\text{R}}\right]$ and virus-specific cytotoxic memory T cells at sites of vaccine administration $\left[\text{mCT}L_{\text{R}}\left(\text{c}\right)\right]$ , and decrease in $\left[\text{mCT}L_{\text{L}}\right]$ upon migration of $\left[\text{mCT}L_{\text{L}}\right]$ toward sites of infection and vaccine administration.
15b	The increase in vaccine-mediated cytotoxic memory T cells at lymph nodes $\left[{\text{mCTL}}_{\text{L}}^{\text{vac}}\right]$ upon transformation of $\left[\text{CD}8^{+}{\text{T}}_{o}\right]$ , natural death of $\left[{\text{mCTL}}_{\text{L}}^{\text{vac}}\right]$ , increase in $\left[{\text{mCTL}}_{\text{L}}^{\text{vac}}\right]$ with migration of vaccine-mediated cytotoxic memory T cells at sites of vaccine administration $\left[{\text{mCTL}}_{\text{r}}^{\text{vac}}\right]$ and vaccine-mediated cytotoxic memory T cells at sites of infection $\left[{\text{mCTL}}_{\text{R}}^{\text{vac}}\right]$ , and decrease in $\left[{\text{mCTL}}_{\text{L}}^{\text{vac}}\right]$ upon migration of $\left[{\text{mCTL}}_{\text{L}}^{\text{vac}}\right]$ toward sites of vaccine administration and infection.
16a	The increase in $\left[\text{mCT}L_{\text{R}}\right]$ with migration of $\left[\text{mCT}L_{\text{L}}\right]$ , decrease in $\left[\text{mCT}L_{\text{R}}\right]$ with migration of $\left[\text{mCT}L_{\text{R}}\right]$ toward lymph nodes, and natural death of $\left[\text{mCT}L_{\text{R}}\right]$ .
16b	The increase in $\left[{\text{mCTL}}_{\text{R}}^{\text{vac}}\right]$ with migration of $\left[{\text{mCTL}}_{\text{L}}^{\text{vac}}\right]$ , decrease in $\left[{\text{mCTL}}_{\text{R}}^{\text{vac}}\right]$ with migration of $\left[\text{mCT}L_{\text{R}}\right]$ toward lymph nodes, and natural death of $\left[{\text{mCTL}}_{\text{R}}^{\text{vac}}\right]$ .
16c	The increase in $\left[{\text{mCTL}}_{\text{L}}^{\text{vac}}\right]$ with migration of $\left[\text{mCT}L_{\text{L}}\right]$ , decrease in $\left[\text{mCT}L_{\text{R}}\right]$ with migration of $\left[\text{mCT}L_{\text{R}}\right]\;$ toward lymph nodes, and natural death of $\left[\text{mCT}L_{\text{R}}\right]$ .
16d	The increase in $\left[{\text{mCTL}}_{\text{L}}^{\text{vac}}\left(\text{c}\right)\right]$ with migration of $\left[{\text{mCTL}}_{\text{L}}^{\text{vac}}\right]$ , decrease in $\left[{\text{mCTL}}_{\text{R}}^{\text{vac}}\left(\text{c}\right)\right]$ with migration of $\left[{\text{mCTL}}_{\text{R}}^{\text{vac}}\left(\text{c}\right)\right]$ toward lymph nodes, and natural death of $\left[{\text{mCTL}}_{\text{R}}^{\text{vac}}\left(\text{c}\right)\right]$ .
17a	The increase in [Tfh] by transformation of $\left[\text{CD}4^{+}{\text{T}}_{o}\right]$ and decrease with natural death of [Tfh] .
17b	The increase in $\left[\text{Tf}{\text{h}}^{\text{vac}}\right]$ by transformation of $\left[\text{CD}4^{+}{\text{T}}_{o}\right]$ and decrease in $\left[\text{Tf}{\text{h}}^{\text{vac}}\right]$ with the natural death of $\left[\text{Tf}{\text{h}}^{\text{vac}}\right]$ .
18	The regeneration and natural death of naïve B cells $\left[{\text{B}}_{\text{o}}\right]$ , decrease in $\left[{\text{B}}_{\text{o}}\right]$ due to differentiation into virus-specific short-lived plasma B cells [pBS] , virus-specific long-lived plasma B cells [PBL] , virus-specific memory B cells at lymph nodes $\left[\text{m}{\text{B}}_{\text{L}}\right]$ , vaccine-mediated short-lived plasma B cells $\left[\text{pB}{\text{S}}^{\text{vac}}\right]$ , vaccine-mediated long-lived plasma B cells $\left[\text{pB}{\text{L}}^{\text{vac}}\right]$ , and vaccine-mediated memory B cells at lymph nodes $\left[{\text{mB}}_{\text{L}}^{\text{vac}}\right]$ .
19a	The increase in [pBS] from differentiation of $\left[{\text{B}}_{\text{o}}\right]$ and decrease in [pBS] with the natural death of [pBS] .
19b	The increase in $\left[{\text{pB}}_{\text{S}}^{\text{vac}}\right]$ from differentiation of $\left[{\text{B}}_{\text{o}}\right]$ and decrease in $\left[{\text{pB}}_{\text{S}}^{\text{vac}}\right]$ with the natural death of $\left[{\text{pB}}_{\text{S}}^{\text{vac}}\right]$ .
20a	The increase in [pBL] by differentiation of $\left[{\text{B}}_{\text{o}}\right]$ , transformation of virus-specific memory B cells at sites of infection $\left[\text{m}{\text{B}}_{\text{R}}\right]$ , virus-specific memory B cells at sites of vaccine administration $\left[\text{m}{\text{B}}_{\text{R}}\left(\text{c}\right)\right]$ , and decrease in [pBL] with the natural death of [pBL] .
20b	The increase in $\left[\text{pB}{\text{L}}^{\text{vac}}\right]$ by differentiation of $\left[{\text{B}}_{\text{o}}\right]$ , transformation of vaccine-mediated memory B cells at sites of vaccine administration $\left[{\text{mB}}_{\text{R}}^{\text{vac}}\right]$ , vaccine-mediated memory B cells at sites of infection $\left[{\text{mB}}_{\text{L}}^{\text{vac}}\left(\text{c}\right)\right]$ , and decrease in $\left[\text{pB}{\text{L}}^{\text{vac}}\right]$ with the natural death of $\left[\text{pB}{\text{L}}^{\text{vac}}\right]$ .
21a	The increase in virus-specific immunoglobulin [Iɡ] produced by [pBS] and [pBL] , decrease in [Iɡ] due to the natural degradation of [Iɡ] , binding of [Iɡ] to viruses and spike protein antigens.
21b	The increase in vaccine-mediated immunoglobulin [Iɡ] produced by $\left[\text{pB}{\text{S}}^{\text{vac}}\right]$ and $\left[\text{pB}{\text{SL}}^{\text{vac}}\right]$ , decrease in $\left[\text{I}{\text{ɡ}}^{\text{vac}}\right]$ due to the natural degradation of $\left[\text{I}{ɡ}^{\text{vac}}\right]$ , binding of $\left[\text{I}{ɡ}^{\text{vac}}\right]$ to spike protein antigens and viruses.
22a	The increase in $\left[\text{m}{\text{B}}_{\text{L}}\right]$ due to differentiation of $\left[{\text{B}}_{\text{o}}\right]$ , migration of $\left[\text{m}{\text{B}}_{\text{R}}\right]$ and $\left[\text{m}{\text{B}}_{\text{R}}\left(\text{c}\right)\right]$ toward lymph nodes, and decrease in $\left[\text{m}{\text{B}}_{\text{L}}\right]$ due to the migration of $\left[\text{m}{\text{B}}_{\text{L}}\right]$ toward sites of infection and vaccine administration, and natural death of $\left[\text{m}{\text{B}}_{\text{L}}\right]$ .
22b	The increase in $\left[{\text{mB}}_{\text{L}}^{\text{vac}}\right]$ due to differentiation of $\left[{\text{B}}_{\text{o}}\right]$ , migration of $\left[{\text{mB}}_{\text{L}}^{\text{vac}}\right]$ and $\left[\text{m}{\text{mB}}_{\text{R}}^{\text{vac}}\left(\text{c}\right)\right]$ toward lymph nodes, and decrease in $\left[{\text{mB}}_{\text{L}}^{\text{vac}}\right]$ due to migration of $\left[{\text{mB}}_{\text{L}}^{\text{vac}}\right]$ toward sites of infection and vaccine administration, and natural death of $\left[{\text{mB}}_{\text{L}}^{\text{vac}}\right]$ .
23a	The increase in $\left[\text{m}B_{\text{R}}\right]$ due to migration of $\left[\text{m}{\text{B}}_{\text{L}}\right]$ and decrease in $\left[\text{m}{\text{B}}_{\text{R}}\right]$ due to migration of $\left[\text{m}{\text{B}}_{\text{R}}\right]$ toward lymph nodes, transformation of $\left[\text{m}{\text{B}}_{\text{R}}\right]$ into [pBL] , and natural death of $\left[\text{m}B_{\text{R}}\right]$ .
23b	The increase in $\left[{\text{mB}}_{\text{R}}^{\text{vac}}\right]$ due to migration of $\left[{\text{mB}}_{\text{L}}^{\text{vac}}\right]$ and decrease in $\left[{\text{mB}}_{\text{R}}^{\text{VAC}}\right]$ due to migration of $\left[{\text{mB}}_{\text{R}}^{\text{vac}}\right]$ toward lymph nodes, transformation of $\left[{\text{mB}}_{\text{R}}^{\text{vac}}\right]$ into $\left[\text{pB}{\text{L}}^{\text{vac}}\right]$ , and natural death of $\left[{\text{mB}}_{\text{R}}^{\text{vac}}\right]$ .
23c	The increase in $\left[\text{m}B_{\text{R}}\left(\text{c}\right)\right]$ due to migration of $\left[\text{m}B_{\text{L}}\right]$ and decrease in $\left[\text{m}B_{\text{R}}\left(\text{c}\right)\right]$ due to migration of $\left[\text{m}{\text{B}}_{\text{R}}\left(\text{c}\right)\right]$ toward lymph nodes, transformation of $\left[\text{m}{\text{B}}_{\text{R}}\left(\text{c}\right)\right]$ into [pBL] , and natural death of $\left[\text{m}{\text{B}}_{\text{R}}\left(\text{c}\right)\right]$ .
23d	The increase in $\left[\text{m}B_{\text{R}}^{\text{vac}}\left(\text{c}\right)\right]$ due to migration of $\left[\text{m}B_{\text{L}}^{\text{vac}}\right]$ and decrease in $\left[\text{m}B_{\text{R}}^{\text{vac}}\left(\text{c}\right)\right]$ due to migration of $\left[\text{m}B_{\text{R}}^{\text{vac}}\left(\text{c}\right)\right]$ toward lymph nodes, transformation of $\left[\text{m}B_{\text{R}}^{\text{vac}}\left(\text{c}\right)\right]\;$ into $\left[p\text{B}{\text{L}}^{\text{vac}}\right]$ , and natural death of $\left[\text{m}B_{\text{R}}^{\text{vac}}\left(\text{c}\right)\right]$ .
5ab	The virus replication by infected cells that are suppressed by antiviral drug [3CLPI] , natural death of [V] , decrease in [V] upon the viral infection to healthy cells, phagocytosis of [V] by dendritic cells, and neutralization of [V] by antibodies.
24	The influx of antiviral drug [3CLPI] and natural degradation of [3CLPI] .

### Simulations

The ODEs comprising a mathematical model of the immune response to SARS-CoV-2 infection and vaccination were solved using the LSODA solver in the COPASI biochemical system simulator (v. 4.37) (110) to obtain variable and flux time courses. The timestep required to solve the ODEs is automatically selected by the integrator in the LSODA solver. The initial concentrations and model parameters used in the simulations are summarized in **Supplementary Tables S1**, **S2** (see the SM), respectively. The baseline model parameters listed in **Supplementary Tables S1**, **S2** without references were adjusted such that the baseline model simulation reproduced clinically observed data, for example, antibody titer [Ig^vac^] upon primary vaccination series (**Figure 3A**), viral load [V] for patients without vaccination upon infection (**Figure 4A**), and antibody titer [Ig] in symptomatic patients (**Figure 4B**). Here, a literature value was employed as the initial estimated parameter, if available from the existing literature, including our previous work. Consequently, we confirmed that the baseline simulation was consistent with the antibody titers [Ig] clinically observed in seropositive individuals in the primary vaccine series (**Figure 5A**). Steady-state solution determination and linear stability analyses were performed using COPASI (110).

## Data Availability

The raw data supporting the conclusions of this article will be made available by the authors, without undue reservation.
