# Supplementary material for: Vaccine and antiviral drug promise for preventing post-acute sequelae of COVID-19, and their combination for its treatment
Source: Front Immunol. 2024 Aug 9;15:1329162. doi: 10.3389/fimmu.2024.1329162 (PMC11341427; doi:10.3389/fimmu.2024.1329162)
Supplement: Supplementary file 1 [file DataSheet_1.pdf]

Supplementary material for

Vaccine and antiviral drug promise for preventing post-acute sequelae of COVID-19, and their combination for its treatment

Tomonari Sumi[1,2] and Kouji Harada[3,4]

[1] Research Institute for Interdisciplinary Science, Okayama University, 3-1-1 Tsushima-Naka, Kita-ku, Okayama 700-8530, Japan

[2] Department of Chemistry, Faculty of Science, Okayama University, 3-1-1 Tsushima-Naka, Kita-ku, Okayama 700-8530, Japan

[3] Department of Computer Science and Engineering, Toyohashi University of Technology, Tempaku-cho, Toyohashi 441-8580, Japan

[4] Center for IT-Based Education, Toyohashi University of Technology, Tempaku-cho, Toyohashi, Aichi, 441-8580, Japan

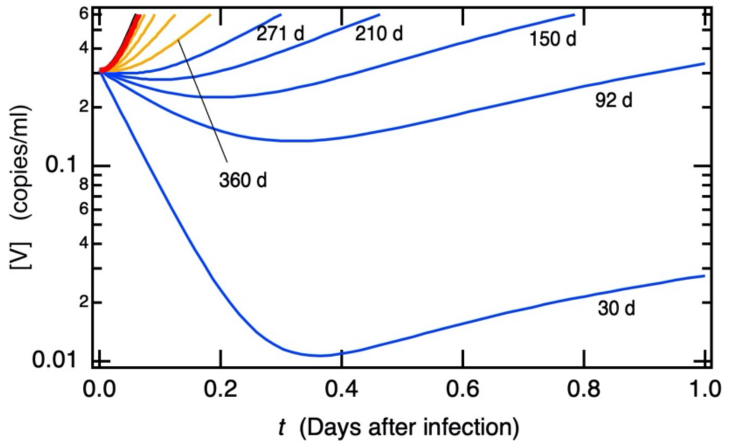

Figure S1. Time courses of [V] around the first minimum in the model with $\theta_{cross} = 1.0$ by varying infection timing from 2nd vaccine dose.

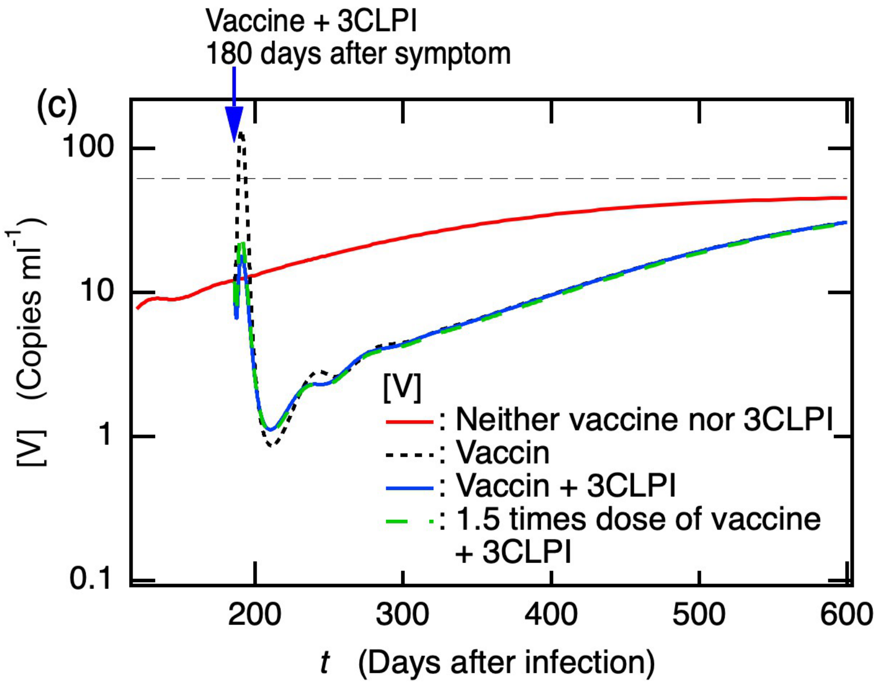

Figure S2. Time course of [V] for patient with a 1.5-fold higher dose of vaccine and 3CLPI administration 180 days after symptom onset. For comparison, the cases for patients with the normal dose of vaccine and 3CLPI administration, with only the normal dose of vaccine, and with neither vaccination nor 3CLPI administration are also shown.

Linear stability analysis based on the eigenvalues of the Jacobian matrix

Table A1. The steady state of the baseline model with zero value of [V], related to the information for Figure 4. The fixed point for [V] = 0 is unstable in the baseline model.

Type of eigenvalue	Number
Purely real	44
Purely imaginary	0
Complex	0
Zero value	2
Positive real part	1
Negative real part	43

Table A2. The steady state of the baseline model with a finite value of [V], related to the information for Figure 4. The steady state with the finite value of [V] is asymptotically stable. The steady state value of [V] for persons without pre-vaccination is 48.1874 copies ml^{-1}.

Type of eigenvalue	Number
Purely real	42
Purely imaginary	0
Complex	4
Zero value	0
Positive real part	0
Negative real part	46

Table A3. Parameter dependent stability of the steady state with [V] = 0, related to the information for Figures 4. The viral infection rate π_I and viral production rate π_V are reduced from the baseline model (**Table 1**, $\boldsymbol{\pi_I}$ = 2.0×10^{-6} day^{-1} ml $copies^{-1}$, $\boldsymbol{\pi_V}$ = 700 day^{-1} copies $cells^{-1}$).

Varying viral infection rate $\boldsymbol{\pi_I}$ (day^{-1} ml $copies^{-1}$)	Varying production rate of virus $\boldsymbol{\pi_V}$ (day^{-1} copies $cells^{-1}$)	Linear stability analysis of the steady state for [V] = 0
$2.0 \times 10^{-6} \times 0.1$	700×0.1	Unstable
$2.0 \times 10^{-6} \times 0.01$	700×0.01	Unstable
$2.0 \times 10^{-6} \times 0.001$	700×0.001	Stable

Table A4. The steady state value of [V] obtained from the baseline model (no vaccinations) and that without the memory T and B cells, related to the information for Figures 4.

Model	Steady state [V] (Copies ml^{-1})	Linear stability analysis
Baseline model (no vaccinations)	48.19	Stable
Baseline model (no vaccinations) without the transformation of $CD8^{+}T_0$ to $mCTL_L$	48.19	Stable
Baseline model (no vaccinations) without the transformation of B_0 to mB_L	48.66	Stable
Baseline model (no vaccinations) without the effects of both the memory T and B cells	48.66	Stable

Table A5. Vaccination effect on the steady state value of [V] and the linear stability in seropositive patient with persistent infection, related to the information for Figure 5.

Timing of vaccine dose after infection	Steady state [V] (Copies ml^{-1})	Linear stability analysis
180 days	48.37	Stable
360 days	48.43	Stable
No vaccination	48.19	Stable

Table A6. Virus co-existence steady state value of [V] and the linear stability for patient who was infected 30, 271, and 722 days after 2nd dose of primary vaccination series, related to the information for Figures 6.

Timing of infection after 2nd dose of primary vaccination series	Steady state [V] (Copies ml^{-1})	Linear stability analysis
30 days	48.18	Stable
271 days	48.18	Stable
722 days	48.18	Stable

Table S1. Definition of the variables in the model.

Symbol	Definition	Initial value
$[\boldsymbol{H}]$	Population of susceptible healthy cells.	4.0×10^5 cells ml^{-1} (1)
$[\boldsymbol{I}]$	Population of infected cells.	0 cells ml^{-1}
$[\boldsymbol{H^{vac}}]$	Population of host cells that have taken up vaccine mRNA.	0 cells ml^{-1}
$[\boldsymbol{DC}]$	Population of dendritic cells.	1.0×10^3 cells ml^{-1} (2)
$[\boldsymbol{APC_R}]$	Population of viral antigen-presenting cells at sites of infection.	0 cells ml^{-1}
$[\boldsymbol{APC_R^{vac}}]$	Population of vaccine-mediated antigen-presenting cells at sites of vaccine administration.	0 cells ml^{-1}
$[\boldsymbol{V}]$	The viral load of free SARS-CoV-2.	0.31 copies ml^{-1} (3)
$[\boldsymbol{S}]$	The fold change of spike proteins of SARS-CoV-2.	0 molecules ml^{-1}
$[\boldsymbol{APC_L}]$	Population of viral antigen-presenting cells at lymph nodes.	0 cells ml^{-1}
$[\boldsymbol{APC_L^{vac}}]$	Population of vaccine-mediated antigen-presenting cells at lymph nodes.	0 cells ml^{-1}
$[\boldsymbol{CD4^+T_o}]$	Population of naïve $CD4^+$ T cells.	1.0×10^3 cells ml^{-1} (2)
$[\boldsymbol{Th1}]$	Population of virus-specific type I helper T cells.	0 cells ml^{-1}
$[\boldsymbol{Th1^{vac}}]$	Population of vaccine-mediated type I helper T cells.	0 cells ml^{-1}
$[\boldsymbol{CD8^+T_o}]$	Population of naïve $CD8^+$ T cells.	1.0×10^3 cells ml^{-1} (2)
$[\boldsymbol{CTL_L}]$	Population of virus-specific cytotoxic T lymphocyte at lymph nodes.	0 cells ml^{-1}
$[\boldsymbol{CTL_L^{vac}}]$	Population of vaccine-mediated cytotoxic T lymphocyte at lymph nodes.	0 cells ml^{-1}
$[\boldsymbol{CTL_R}]$	Population of virus-specific cytotoxic T lymphocyte at sites of infection.	0 cells ml^{-1}
$[\boldsymbol{CTL_R^{vac}}]$	Population of vaccine-mediated cytotoxic T lymphocyte at sites of vaccine administration.	0 cells ml^{-1}
$[\boldsymbol{CTL_R(c)}]$	Population of virus-specific cytotoxic T lymphocyte at sites of vaccine administration.	0 cells ml^{-1}
$[\boldsymbol{CTL_R^{vac}(c)}]$	Population of vaccine-mediated cytotoxic T lymphocyte at sites of infection.	0 cells ml^{-1}
$[\boldsymbol{Tfh}]$	Population of virus-specific follicular helper T cells.	0 cells ml^{-1}
$[\boldsymbol{Tfh^{vac}}]$	Population of vaccine-mediated follicular helper T cells.	0 cells ml^{-1}
$[\boldsymbol{B_o}]$	Population of naïve B cells.	1.0×10^3 cells ml^{-1} (2)
$[\boldsymbol{pBS}]$	Population of virus-specific short-lived plasma B cells.	0 cells ml^{-1}
$[\boldsymbol{pBS^{vac}}]$	Population of vaccine-mediated short-lived plasma B cells.	0 cells ml^{-1}
$[\boldsymbol{pBL}]$	Population of virus-specific long-lived plasma B cells.	0 cells ml^{-1}
$[\boldsymbol{pBL^{vac}}]$	Population of vaccine-mediated long-lived plasma B cells.	0 cells ml^{-1}
$[\boldsymbol{mCTL_L}]$	Population of virus-specific cytotoxic memory T cells at lymph nodes.	0 cells ml^{-1}
$[\boldsymbol{mCTL_L^{vac}}]$	Population of vaccine-mediated cytotoxic memory T cells at lymph nodes.	0 cells ml^{-1}
$[\boldsymbol{mCTL_R}]$	Population of virus-specific cytotoxic memory T	0 cells ml^{-1}

	cells at sites of infection.	
$[\boldsymbol{mCTL_R^{vac}}]$	Population of vaccine-mediated cytotoxic memory T cells at sites of vaccine administration.	0 cells ml^{-1}
$[\boldsymbol{mCTL_R(c)}]$	Population of virus-specific cytotoxic memory T cells at sites of vaccine administration.	0 cells ml^{-1}
$[\boldsymbol{mCTL_R^{vac}(c)}]$	Population of vaccine-mediated cytotoxic memory T cells at sites of infection.	0 cells ml^{-1}
$[\boldsymbol{mB_L}]$	Population of virus-specific memory B cells at lymph nodes.	0 cells ml^{-1}
$[\boldsymbol{mB_L^{vac}}]$	Population of vaccine-mediated memory B cells at lymph nodes.	0 cells ml^{-1}
$[\boldsymbol{mB_R}]$	Population of virus-specific memory B cells at sites of infection.	0 cells ml^{-1}
$[\boldsymbol{mB_R^{vac}}]$	Population of vaccine-mediated memory B cells at sites of vaccine administration.	0 cells ml^{-1}
$[\boldsymbol{mB_R(c)}]$	Population of virus-specific memory B cells at sites of vaccine administration.	0 cells ml^{-1}
$[\boldsymbol{mB_R^{vac}(c)}]$	Population of vaccine-mediated memory B cells at sites of infection.	0 cells ml^{-1}
$[\boldsymbol{Ig}]$	Antibody titer for virus-specific immunoglobulin.	110 titer (2)
$[\boldsymbol{Ig^{vac}}]$	Antibody titer for vaccine-mediated immunoglobulin.	0 titer
$[\boldsymbol{INF1}]$	The concentration of type-I interferon.	0 molecules ml^{-1}
$[\boldsymbol{INF1^{vac}}]$	The concentration of vaccine-mediated INF1.	0 molecules ml^{-1}
$[\boldsymbol{CXCL}]$	The concentration of CXC chemokine receptor ligand (CXCL).	0 molecules ml^{-1}
$[\boldsymbol{CXCL^{vac}}]$	The concentration of vaccine-mediated CXCL.	0 molecules ml^{-1}
$[\boldsymbol{Vac}]$	The concentration of liposomes containing vaccine mRNA.	0 liposomes ml^{-1}
$[\boldsymbol{3CLPI}]$	The concentration of 3C-like protease inhibitor.	0 mg ml^{-1}

Table S2. Definition of the parameters in the model.

Symbol	Definition	Values	References
$\boldsymbol{\lambda_H}$	Supply rate of susceptible healthy epithelial cells.	4.0×10^{3} cells ml^{-1} day^{-1}	(1)
$\boldsymbol{\delta_H}$	Natural death rate of susceptible healthy epithelial cells.	1.0×10^{-2} day^{-1}	(1)
$\boldsymbol{\lambda_{DC}}$	Supply rate of dendritic cells.	1.0×10^{1} cells ml^{-1} day^{-1}	(1)
$\boldsymbol{\delta_{DC}}$	Natural death rate of dendritic cells.	1.0×10^{-2} day^{-1}	(1)
$\boldsymbol{\lambda_{CD4}}$	Supply rate of naïve $CD4^{+}$ T cells.	2.0×10^{1} cells ml^{-1} day^{-1}	(1)
$\boldsymbol{\delta_{CD4}}$	Natural death rate of naïve $CD4^{+}$ T cells.	2.0×10^{-2} day^{-1}	(4,5)
$\boldsymbol{\lambda_{CD8}}$	Supply rate of naïve $CD8^{+}$ T cells.	2.0×10^{1} cells ml^{-1} day^{-1}	(1)
$\boldsymbol{\delta_{CD8}}$	Natural death rate of naïve $CD8^{+}$ T cells.	2.0×10^{-2} day^{-1}	(4,5)
$\boldsymbol{\lambda_B}$	Supply rate of naïve B cells.	2.0×10^{2} cells ml^{-1} day^{-1}	(1)
$\boldsymbol{\delta_B}$	Natural death rate of naïve B cells.	2.0×10^{-1} day^{-1}	(4,6)
$\boldsymbol{\pi_I}$	Infection rate of susceptible healthy epithelial cells.	2.0×10^{-6} day^{-1} ml $copies^{-1}$	(1)
$\boldsymbol{\beta_I}$	Antibody neutralization rate.	5.0×10^{-3} ml $copies^{-1}$ $titer^{-1}$	(1)
$\boldsymbol{\theta_{cross}}$	Reduction rate for several cross reactions, e.g., between viral infection and vaccination.	0.8	
$\boldsymbol{\delta_I}$	Natural death rate of infected cells.	1.0×10^{-2} day^{-1}	δ_I is much smaller than δ_V. (7)
$\boldsymbol{k_{I\_CTL}}$	Rate of killing of infected cells by virus-specific cytotoxic T lymphocyte.	3.0×10^{-2} day^{-1} ml $cells^{-1}$	(1)
$\boldsymbol{k_{I\_mCTL}}$	Rate of killing of infected cells by virus-specific cytotoxic memory T cells.	8.0×10^{-10} day^{-1} $(ml\ cells^{-1})^2$ $copies^{-1}$ ml	
$\boldsymbol{k_{I\_mCTL}^{vac}}$	Rate of killing of host cells with taken up vaccine mRNA by vaccine-mediated cytotoxic memory T cells.	3.3×10^{-11} day^{-1} $(ml\ cells^{-1})^2$ $molecules^{-1}$ ml	
$\boldsymbol{\pi_{vac}}$	Uptake rate of vaccine mRNA by healthy epithelial cells.	2.0×10^{-5} day^{-1} ml $liposomes^{-1}$	Assumed to be similar to π_I.
$\boldsymbol{\delta_I^{vac}}$	Natural death rate of host cells with taken up vaccine mRNA.	1.0×10^{-2} day^{-1}	Assumed to be similar to δ_I.
$\boldsymbol{k_{I\_CTL}^{vac}}$	Rate of killing of host cells with taken up vaccine mRNA by vaccine-mediated cytotoxic T lymphocyte.	3.0×10^{-2} day^{-1} ml $cells^{-1}$	Assumed to be similar to k_{I_CTL}.
$\boldsymbol{\pi_{APC}}$	Uptake of virus and viral antigen-presenting rate of dendritic cells.	3.0×10^{-6} day^{-1} ml $copies^{-1}$	(1)
$\boldsymbol{\pi_{APC}^{vac}}$	Uptake of vaccine-mediated spike proteins and antigen-presenting rate of dendritic cells.	2.8×10^{-9} day^{-1} ml $molecules^{-1}$	
$\boldsymbol{\alpha_{recruit}}$	Recruitment efficiency of DC/CTL by type-I interferon.	1.0×10^{-3} $molecules^{-1}$ ml	(1)
$\boldsymbol{\alpha_{APC}}$	Regulation of viral antigen-presenting rate of dendritic cells by Ig.	3.0×10^{-2} $titer^{-1}$	(1)

α_{APC}^{vac}	Regulation of vaccine-mediated antigen-presenting rate of dendritic cells by Ig^{vac}.	6.0×10^{-2} titer^{-1}	Assumed to be equal to α_{APC}.
δ_{APC_R}	Natural death rate of viral antigen-presenting cells at sites of infection.	0.1 day^{-1}	(1)
$\delta_{APC_R}^{vac}$	Natural death rate of vaccine-mediated antigen-presenting cells at sites of vaccine administration.	0.1 day^{-1}	Assumed to be equal to δ_{APC_R}.
μ_{APC}	Migration rate of viral antigen-presenting cells from sites of infection to lymph nodes.	0.2 day^{-1}	(1)
μ_{APC}^{vac}	Migration rate of vaccine-mediated antigen-presenting cells from sites of vaccine administration to lymph nodes.	0.2 day^{-1}	Assumed to be similar to μ_{APC}.
π_V	Production rate of virus by infected cells.	700 day^{-1} copies cells^{-1}	(1)
π_S	Production rate of spike proteins by host cells that have taken up vaccine mRNA.	4670 day^{-1} molecules cells^{-1}	
β_V	Inhibition rate of viral production by type-I interferon.	1.0×10^{-3} molecules^{-1} ml	(1)
β_S	Inhibition rate of spike protein production by type-I interferon.	1.0×10^{-3} molecules^{-1} ml	Assumed to be similar to β_V.
δ_V	Clearance rare of virus.	0.56 day^{-1}	(8)
δ_S	Clearance rare of spike protein.	~~1.40~~ 1,90 day^{-1}	
γ_{Ig}	Neutralized rate of virus by virus-specific Ig.	9.0×10^{-6} day^{-1} titer^{-1}	(1)
γ_{Ig}^{vac}	Neutralized rate of spike protein by vaccine-mediated Ig.	9.0×10^{-7} day^{-1} titer^{-1} or day^{-1} molecules^{-1} ml	
δ_{APC_L}	Natural death rate of viral antigen-presenting cells at lymph nodes.	0.1 day^{-1}	(1)
$\delta_{APC_L}^{vac}$	Natural death rate of vaccine-mediated antigen-presenting cells at lymph nodes.	0.1 day^{-1}	Assumed to be similar to δ_{APC_L}.
π_{Th1}	Differentiation rate of naïve $CD4^+$ T cells into virus-specific type I helper T cells.	6.0×10^{-6} day^{-1} ml cells^{-1}	(1)
π_{Th1}^{vac}	Differentiation rate of naïve $CD4^+$ T cells into vaccine-mediated type I helper T cells.	12.0×10^{-6} day^{-1} ml cells^{-1}	Assumed to be similar to π_{Th1}.
α_{Th1}	Regulation of $CD4^+$ T cell differentiation rate into virus-specific Th1 cells by type I interferon.	1.0×10^{-4} molecules^{-1} ml	(1)
α_{Th1}^{vac}	Regulation of $CD4^+$ T cell differentiation rate into vaccine-mediated Th1 cells by type I interferon.	1.0×10^{-4} molecules^{-1} ml	Assumed to be similar to α_{Th1}.
π_{Tfh}	Differentiation rate of naïve $CD4^+$ T cells into virus-specific follicular helper T cells.	5.0×10^{-5} day^{-1} ml cells^{-1}	(1)
π_{Tfh}^{vac}	Differentiation rate of naïve $CD4^+$ T cells into vaccine-mediated follicular helper T cells.	12.0×10^{-5} day^{-1} ml cells^{-1}	Assumed to be similar to π_{Tfh}.

$\boldsymbol{\alpha_{Tfh}}$	Regulation of CD4+ T cell differentiation rate into virus-specific Tfh cells by type I interferon.	5.0×10^{-4} molecules^{-1} ml	(1)
$\boldsymbol{\alpha^{vac}_{Tfh}}$	Regulation of CD4+ T cell differentiation rate into vaccine-mediated Tfh cells by type I interferon.	5.0×10^{-4} molecules^{-1} ml	Assumed to be similar to α_{Tfh}.
$\boldsymbol{\delta_{Th1}}$	Natural death rate of virus-specific Th1 cells.	0.4 day^{-1}	(2)
$\boldsymbol{\delta^{vac}_{Th1}}$	Natural death rate of vaccine-mediated Th1 cells.	0.4 day^{-1}	Assumed to be similar to δ_{Th1}.
$\boldsymbol{\delta_{Tfh}}$	Natural death rate of virus-specific Tfh cells.	0.4 day^{-1}	(2)
$\boldsymbol{\delta^{vac}_{Tfh}}$	Natural death rate of vaccine-mediated Tfh cells.	0.4 day^{-1}	Assumed to be similar to δ_{Tfh}.
$\boldsymbol{\pi_{CTL}}$	Transformation rate of virus-specific cytotoxic T lymphocyte from naïve CD8+ T cells.	1.0×10^{-4} day^{-1} ml^2 cells^{-2}	(1)
$\boldsymbol{\pi^{vac}_{CTL}}$	Transformation rate of vaccine-mediated cytotoxic T lymphocyte from naïve CD8+ T cells.	2.0×10^{-4} day^{-1} ml^2 cells^{-2}	Assumed to be similar to π_{CTL}.
$\boldsymbol{\delta_{CTL}}$	Natural death rate of virus-specific CTL cells.	0.1 day^{-1}	(9)
$\boldsymbol{\delta^{vac}_{CTL}}$	Natural death rate of vaccine-mediated CTL cells.	0.1 day^{-1}	Assumed to be similar to δ_{CTL}.
$\boldsymbol{\mu_{CTL}}$	Migration rate of virus-specific CTL cells from lymph nodes to sites of infection/vaccine administration.	1.2 day^{-1}	(9)
$\boldsymbol{\mu^{vac}_{CTL}}$	Migration rate of vaccine-mediated CTL cells from lymph nodes to sites of vaccine administration/viral infection.	1.2 day^{-1}	Assumed to be similar to μ_{CTL}.
$\boldsymbol{\omega_{recruit}}$	Recruitment efficiency of CTL/mCTL/mB from lymph nodes to sites of infection/vaccine administration by CXCL.	1.0×10^{-2} molecules^{-1} ml	
$\boldsymbol{\pi_{mCTL}}$	Transformation rate of virus-specific cytotoxic memory T cell from naïve CD8+ T cells.	1.2×10^{-6} day^{-1} ml^2 cells^{-2}	Assumed to be 0.015 times of π_{CTL}.
$\boldsymbol{\pi^{vac}_{mCTL}}$	Transformation rate of vaccine-mediated cytotoxic memory T cell from naïve CD8+ T cells.	2.4×10^{-6} day^{-1} ml^2 cells^{-2}	Assumed to be similar to π_{mCTL}.
$\boldsymbol{\mu_{mCTLL}}$	Migration rate of virus-specific memory CTL cells from sites of infection/vaccine administration to lymph nodes.	0.6 day^{-1}	
$\boldsymbol{\mu^{vac}_{mCTLL}}$	Migration rate of vaccine-mediated memory CTL cells from sites of vaccine administration/infection to lymph nodes.	0.6 day^{-1}	Assumed to be similar to μ_{mCTLL}.
$\boldsymbol{\delta_{mCTL}}$	Natural death rate of virus-specific memory CTL cells.	9.0×10^{-3} day^{-1}	
$\boldsymbol{\delta^{vac}_{mCTL}}$	Natural death rate of vaccine-mediated memory CTL cells.	9.0×10^{-3} day^{-1}	Assumed to be similar to δ_{mCTL}.

$\boldsymbol{\mu_{mCTLR}}$	Migration rate of virus-specific memory CTL cells from lymph nodes to sites of infection/vaccine administration.	1.2 day^{-1}	
$\boldsymbol{\mu_{mCTLR}^{vac}}$	Migration rate of vaccine-mediated memory CTL cells from lymph nodes to sites of vaccine administration/infection.	1.2 day^{-1}	Assumed to be similar to μ_{mCTLR}.
$\boldsymbol{\pi_{pBS}}$	Differentiation rate of naïve B cells into virus-specific short-lived plasma B cells.	8.0 × 10^{-7} day^{-1} ml^2 cells^{-2}	(1)
$\boldsymbol{\pi_{pBS}^{vac}}$	Differentiation rate of naïve B cells into vaccine-mediated short-lived plasma B cells.	16.0 × 10^{-7} day^{-1} ml^2 cells^{-2}	Assumed to be similar to π_{pBS}.
$\boldsymbol{\pi_{pBL}}$	Differentiation rate of naïve B cells into virus-specific long-lived plasma B cells.	2.4 × 10^{-7} day^{-1} ml^2 cells^{-2}	
$\boldsymbol{\pi_{pBL}^{vac}}$	Differentiation rate of naïve B cells into vaccine-mediated long-lived plasma B cells.	4.8 × 10^{-7} day^{-1} ml^2 cells^{-2}	Assumed to be similar to π_{pBL}.
$\boldsymbol{\delta_{pBS}}$	Natural death rate of virus-specific short-lived plasma B cells.	0.1 day^{-1}	(2)
$\boldsymbol{\delta_{pBS}^{vac}}$	Natural death rate of vaccine-mediated short-lived plasma B cells.	0.1 day^{-1}	Assumed to be similar to δ_{pBS}.
$\boldsymbol{\delta_{pBL}}$	Natural death rate of virus-specific long-lived plasma B cells.	9.0 x 10^{-3} day^{-1}	
$\boldsymbol{\delta_{pBL}^{vac}}$	Natural death rate of vaccine-mediated long-lived plasma B cells.	9.0 x 10^{-3} day^{-1}	Assumed to be similar to δ_{pBL}.
$\boldsymbol{\pi_{mB\_pBL}}$	Transformation rate of virus-specific mB_R into virus-specific pBL.	14.4 × 10^{-6} day^{-1} ml copies^{-1}	
$\boldsymbol{\pi_{mB\_pBL}^{vac}}$	Transformation rate of vaccine-mediated mB_R into vaccine-mediated pBL.	6.0 × 10^{-6} day^{-1} ml molecules^{-1}	
$\boldsymbol{\pi_{mBL}}$	Transformation rate of virus-specific memory B cell from naïve B cells.	4.0 × 10^{-9} day^{-1} ml^2 cells^{-2}	
$\boldsymbol{\pi_{mBL}^{vac}}$	Transformation rate of vaccine-mediated memory B cell from naïve B cells.	8.0 × 10^{-9} day^{-1} ml^2 cells^{-2}	Assumed to be similar to π_{mBL}.
$\boldsymbol{\mu_{mBL}}$	Migration rate of virus-specific memory B cells from sites of infection/vaccine administration to lymph nodes.	0.6 day^{-1}	Assumed to be equal to μ_{mCTLL}.
$\boldsymbol{\mu_{mBL}^{vac}}$	Migration rate of vaccine-mediated memory B cells from sites of vaccine administration/infection to lymph nodes.	0.6 day^{-1}	Assumed to be equal to μ_{mBL}.
$\boldsymbol{\delta_{mBL}}$	Natural death rate of virus-specific memory B cells at lymph nodes.	1.0 x 10^{-10} day^{-1}	
$\boldsymbol{\delta_{mBL}^{vac}}$	Natural death rate of vaccine-mediated memory B cells at lymph nodes.	1.0 x 10^{-10} day^{-1}	Assumed to be equal to δ_{mBL}.
$\boldsymbol{\mu_{mBR}}$	Migration rate of virus-specific memory B cells from lymph nodes to sites of infection/vaccine	1.2 day^{-1}	This term is supposed to apply to

	administration.		
$\boldsymbol{\mu_{mBR}^{vac}}$	Migration rate of vaccine-mediated memory B cells from lymph nodes to sites of vaccine administration/infection.	1.2 day^{-1}	Assumed to be equal to μ_{mBR}.
$\boldsymbol{\delta_{mBR}}$	Natural death rate of virus-specific memory B cells at sites of infection/vaccine administration.	1.0 x 10^{-10} day^{-1}	Assumed to be equal to δ_{mBL}.
$\boldsymbol{\delta_{mBR}^{vac}}$	Natural death rate of vaccine-mediated memory B cells at sites of vaccine administration/infection.	1.0 x 10^{-10} day^{-1}	Assumed to be equal to δ_{mBR}.
$\boldsymbol{\pi_{IgS}}$	Antibody production rate by virus-specific pBS.	6.2 × 10^1 day^{-1} titer ml^{-1} $cells^{-1}$	
$\boldsymbol{\pi_{IgS}^{vac}}$	Antibody production rate by vaccine-mediated pBS.	16.5 × 10^1 day^{-1} titer ml^{-1} $cells^{-1}$	
$\boldsymbol{\pi_{IgL}}$	Antibody production rate by virus-specific pBL.	2.0 × 10^1 day^{-1} titer ml^{-1} $cells^{-1}$	
$\boldsymbol{\pi_{IgL}^{vac}}$	Antibody production rate by vaccine-mediated pBL.	6.0× 10^1 day^{-1} titer ml^{-1} $cells^{-1}$	
$\boldsymbol{\delta_{Ig}}$	Degradation rate of virus-specific Ig.	0.07 day^{-1}	(10)
$\boldsymbol{\delta_{Ig}^{vac}}$	Degradation rate of vaccine-mediated Ig.	0.07 day^{-1}	Assumed to be equal to δ_{Ig}.
$\boldsymbol{\xi_{Ig}}$	Consumption rate of virus-specific Ig upon Ig-binding to virus.	$N_s \times \gamma_{Ig}$ = 2.16× 10^{-4} day^{-1} ml $copies^{-1}$	(1)
$\boldsymbol{\sigma_{I}}$	Secretion rate of type I interferon by infected cells.	0.01 day^{-1} molecules $cells^{-1}$	(1)
$\boldsymbol{\sigma_{I}^{vac}}$	Secretion rate of type I interferon by host cells with taken up vaccine mRNA.	1 day^{-1} molecules $cells^{-1}$	
$\boldsymbol{\sigma_{APC}}$	Secretion rate of type I interferon by virus-specific APC cells.	10 day^{-1} molecules $cells^{-1}$	(11)
$\boldsymbol{\sigma_{APC}^{vac}}$	Secretion rate of type I interferon by vaccine-mediated APC cells.	10 day^{-1} molecules $cells^{-1}$	Assumed to be equal to σ_{APC}.
$\boldsymbol{\delta_{IFN1}}$	Degradation rate of type I interferon.	0.7 day^{-1}	(11,12)
$\boldsymbol{\sigma_{CXCL}}$	Secretion rate of CXCL by infected cells.	10 day^{-1} molecules $cells^{-1}$	
$\boldsymbol{\sigma_{CXCL}^{vac}}$	Secretion rate of CXCL by host cells with taken up vaccine mRNA.	10 day^{-1} molecules $cells^{-1}$	Assumed to be equal to σ_{CXCL}.
$\boldsymbol{\delta_{CXCL}}$	Degradation rate of CXCL.	0.7 day^{-1}	
$\boldsymbol{\delta_{Vac}}$	Degradation rate of RNA vaccine.	0.56([V]) day^{-1}	
$\boldsymbol{\delta_{3CLPI}}$	Degradation rate of I3CLP.	0.3465 day^{-1}	$t_{1/2}$ is 2 day for 3C-like protease inhibitor 'Xocova' developed by SHIONOGI & CO., LTD.
$\boldsymbol{\beta_{3CLPI}}$	Inhibition rate of viral production by type-I interferon.	2.0 × 10^2 mg^{-1} ml	

Model of vaccine administration

Influx of liposomes containing vaccine mRNA is used to raise [Vac] and is modeled with two sigmoid functions:

$$J_i^{vac}(t - t_i^{vac}) = A_i^{vac}\left\{\frac{1}{1+exp[-a(t-t_i^{vac})]} - \frac{1}{1+exp\left[-a\left(t-(t_i^{vac}+\Delta t)\right)\right]}\right\}. \tag{S1}$$

We used the parameters shown in the table below for the 1st and 2nd dose of primary series and booster dose.

Table S3. Definition of the parameters in the model of vaccine administration (Eq. S1).

1st dose of primary series	Parameter values
A_i^{vac}	100000 liposomes ml^{-1} day^{-1}
a	100 day^{-1}
t_i^{vac}	0.001 day
Δt	0.005 day
2nd dose of primary series	
A_i^{vac}	100000 liposomes ml^{-1} day^{-1}
a	100 day^{-1}
t_i^{vac}	28.001 day
Δt	0.005 day
Booster dose	
A_i^{vac}	50000 liposomes ml^{-1} day^{-1}
a	100 day^{-1}
t_i^{vac}	Arbitrarily day
Δt	0.005 day

Model of 3C-like protease inhibitor (3CLPI) administration

Influx of antiviral drug is used to raise its concentration [I3CLP] and is modeled with five series of two sigmoid functions:

$$J^{3CLPI}(t - t_s) = A_1^{3CLPI}\left\{\frac{1}{1+exp[-a(t-t_s)]} - \frac{1}{1+exp\left[-a\left(t-(t_s+\Delta t)\right)\right]}\right\} + A_2^{3CLPI}\left\{\frac{1}{1+exp[-a(t-(t_s+1))]} - \frac{1}{1+exp\left[-a\left(t-((t_s+1)+\Delta t)\right)\right]}\right\} + A_2^{3CLPI}\left\{\frac{1}{1+exp[-a(t-(t_s+2))]} - \frac{1}{1+exp\left[-a\left(t-((t_s+2)+\Delta t)\right)\right]}\right\} + A_2^{3CLPI}\left\{\frac{1}{1+exp[-a(t-(t_s+3))]} - \frac{1}{1+exp\left[-a\left(t-((t_s+3)+\Delta t)\right)\right]}\right\} + A_2^{3CLPI}\left\{\frac{1}{1+exp[-a(t-(t_s+4))]} - \frac{1}{1+exp\left[-a\left(t-((t_s+4)+\Delta t)\right)\right]}\right\}, \tag{S2}$$

which correspond to the administrations for five days. We used the parameters shown in the table below.

Table S4. Definition of the parameters in the model of 3CLPI administration (Eq. S2).

	Parameter values
A_1^{3CLPI}	40.8 mg ml^{-1} day^{-1}
A_2^{3CLPI}	13.6 mg ml^{-1} day^{-1}
a	100 day^{-1}
t_s	3 day
Δt	0.001 day

REFERENCES

1. Sumi T, Harada K. Immune response to SARS-CoV-2 in severe disease and long COVID-19. *iScience* (2022) **25**:104723. doi:10.1016/j.isci.2022.104723

2. Lee HY, Topham DJ, Park SY, Hollenbaugh J, Treanor J, Mosmann TR, Jin X, Ward BM, Miao H, Holden-Wiltse J, et al. Simulation and prediction of the adaptive immune response to influenza A virus infection. *J Virol* (2009) **83**:7151–7165. doi:10.1128/JVI.00098-09

3. Hernandez-Vargas EA, Velasco-Hernandez JX. In-host Mathematical Modelling of COVID-19 in Humans. *Annu Rev Control* (2020) **50**:448–456. doi:10.1016/j.arcontrol.2020.09.006

4. Oprea M, Perelson AS. Exploring the mechanisms of primary antibody responses to T cell-dependent antigens. *J Theor Biol* (1996) **181**:215–236. doi:10.1006/jtbi.1996.0127

5. Vonboehmer H, Hafen K. The Life-Span of Naive Alpha/Beta T-Cells in Secondary Lymphoid Organs. *J Exp Med* (1993) **177**:891–896. doi:10.1084/jem.177.4.891

6. Chan E, Maclennan I. Only a Small Proportion of Splenic B-Cells in Adults Are Short-Lived Virgin Cells. *Eur J Immunol* (1993) **23**:357–363. doi:10.1002/eji.1830230209

7. Kim KS, Ejima K, Iwanami S, Fujita Y, Ohashi H, Koizumi Y, Asai Y, Nakaoka S, Watashi K, Aihara K, et al. A quantitative model used to compare within-host SARS-CoV-2, MERS-CoV, and SARS-CoV dynamics provides insights into the pathogenesis and treatment of SARS-CoV-2. *PLoS Biol* (2021) **19**:e3001128. doi:10.1371/journal.pbio.3001128

8. Ejima K, Kim KS, Ludema C, Bento AI, Iwanami S, Fujita Y, Ohashi H, Koizumi Y, Watashi K, Aihara K, et al. Estimation of the incubation period of COVID-19 using viral load data. *Epidemics* (2021) **35**:100454. doi:10.1016/j.epidem.2021.100454

9. Zarnitsyna VI, Handel A, McMaster SR, Hayward SL, Kohlmeier JE, Antia R. Mathematical Model Reveals the Role of Memory CD8 T Cell Populations in Recall Responses to Influenza. *Front Immunol* (2016) **7**:165. doi:10.3389/fimmu.2016.00165

10. Nikin-Beers R, Ciupe SM. The role of antibody in enhancing dengue virus infection. *Math Biosci* (2015) **263**:83–92. doi:10.1016/j.mbs.2015.02.004

11. Ghosh I. Within Host Dynamics of SARS-CoV-2 in Humans - Modeling Immune Responses and Antiviral Treatments. *SN Comput Sci* (2021) **2**:482. doi:10.1007/s42979-021-00919-8

12. Harari D, Kuhn N, Abramovich R, Sasson K, Zozulya AL, Smith P, Schlapschy M, Aharoni R,

Köster M, Eilam R, et al. Enhanced in vivo efficacy of a type I interferon superagonist with extended plasma half-life in a mouse model of multiple sclerosis. *J Biol Chem* (2014) **289**:29014–29029. doi:10.1074/jbc.M114.602474
